# Long-read genome sequencing enhances diagnostics of pediatric neurological disorders

**DOI:** 10.1186/s13073-025-01596-5

**Published:** 2026-01-09

**Authors:** Marlene Ek, Malin Kvarnung, Esmee Ten Berk de Boer, Linnéa La Fleur, Lena Ljöstad, Anna Lyander, Søren Lejsted Faergeman, Simon Opstrup Drue, Håkan Thonberg, Ann Nordgren, Maria Johansson Soller, Valtteri Wirta, Jesper Eisfeldt, Anna Lindstrand

**Affiliations:** 1 Department of Molecular Medicine and Surgery, Karolinska Institutet, Stockholm, 171 64 Sweden; 2 Department of Clinical Genetics and Genomics, Karolinska University Hospital, Stockholm, 171 76 Sweden; 3 Science for Life Laboratory, Department of Molecular Medicine and Surgery, Karolinska Institutet, Solna, 171 65 Sweden; 4 Science for Life Laboratory, Department of Microbiology, Tumor and Cell Biology, Karolinska Institutet, Solna, 171 65 Sweden; 5 Science for Life Laboratory, School of Chemistry, Biotechnology and Health, KTH Royal Institute of Technology, Stockholm, 171 65 Sweden; 6 Department of Clinical Genetics, Aarhus University Hospital, Aarhus N, 8200 Denmark; 7 Department of Molecular Medicine, Aarhus University Hospital, Aarhus N, 8200 Denmark; 8 Department of Laboratory Medicine, Institute of Biomedicine, University of Gothenburg, Gothenburg, 405 30 Sweden; 9 Department of Clinical Genetics and Genomics, Sahlgrenska University Hospital, Region Västra Götaland, Gothenburg, 413 45 Sweden; 10 Genomic Medicine Center Karolinska, Karolinska University Hospital, Stockholm, 171 76 Sweden

**Keywords:** Whole genome sequencing, Long-read sequencing, Short-read sequencing, Rare diseases, Clinical diagnostics, Single nucleotide variants, Chromosomal rearrangements, Structural variants, Short tandem repeat expansions, Methylation analysis

## Abstract

**Background:**

Singleton short-read genome sequencing (GS) is increasingly used as a first-line genetic test for childhood neurological disorders (such as intellectual disability, neurodevelopmental delay, motor delay, and hypotonia) with diagnostic yields from 26 to 35%, typically involving a mix of single nucleotide variants and small insertions/deletions (SNV/INDELs), structural variants (SVs), and short tandem repeats (STRs). Long-read GS is emerging as an attractive alternative, offering a more comprehensive assessment of the genome, but its utility still needs to be systematically evaluated in a clinical diagnostic setting.

**Methods:**

We prospectively included 100 children and adolescents (≤ 20 years) with neurological disorders, newly referred for genetic testing. Routine DNA was used for singleton standard clinical short-read GS in parallel with long-read GS (Oxford Nanopore Technologies). In addition to comprehensive variant calling, long-read GS data was also phased and underwent methylation analysis. Variant interpretation was restricted to in-silico gene panels targeting either intellectual disability (1,568 genes) or neuromuscular disorders (1,035 genes) depending on the clinical presentation.

**Results:**

The long-read GS generated an average of 111 GB data per sample, with a median read-length of 5 kb and average N50 of 16 kb; resulting in an average coverage of 34X. Short-read and long-read GS identified the same 29% diagnostic yield, including SNV/INDELs (*n* = 18), SVs (*n* = 9), STRs (*n* = 1), and uniparental disomy (*n* = 1). Long-read GS provided additional diagnostic value in 13 cases involving 17 distinct variants, including phasing of *SMN1* and biallelic SNVs/INDELs in autosomal recessive genes, accurate determination of STR length and sequence as well as detailed structural characterization of SVs. Of note, an unbalanced translocation, der(14)t(8;14)(p11.2;p23.1), required *de novo* assembly and T2T-CHM13 alignment to resolve the breakpoint junctions. Furthermore, long-read GS detected disease-associated aberrant methylation patterns in the Prader-Willi region and across an *FMR1* expansion.

**Conclusions:**

In a clinical diagnostic setting, long-read GS proved to be a streamlined, first-line test, capturing the full spectrum of disease-causing variants, reducing the need for follow-up testing and enabling more precise interpretation. While the overall diagnostic yield may be comparable to that of short-read approaches, long-read GS offers significant added value across multiple variant types.

**Supplementary Information:**

The online version contains supplementary material available at 10.1186/s13073-025-01596-5.

## Background

Neurological disorders encompass a broad spectrum of conditions that affect the central and/or peripheral nervous system. In children and adolescents, these disorders include neurodevelopmental disorders (NDD), such as developmental delay, intellectual disability (ID) and autism, as well as neuromuscular disorders (NMD). Intellectual disability affects approximately 1% of the population and involves limitations in intellectual functioning and adaptive behavior (e.g., language, social and practical skills) [1, 2]. Intellectual disability can occur in isolation, together with other neurocognitive conditions (autism, attention deficit hyperactivity disorder), together with seizures or as part of a broader syndromic presentation [3]. Neuromuscular disorders (NMDs) are a heterogeneous group of disorders affecting the peripheral nerves, muscles, or muscular junction. Hypotonia and delayed motor development are common presentations in children with NMDs. However, there is often a phenotypic overlap in the presentation of NDDs and NMDs, particularly in young children [4].

The etiology of neurological disorders is heterogeneous and may include infections, or environmental factors, but a considerable proportion are genetic (i.e. monogenic or chromosomal) [5, 6]. Using short-read genome sequencing (GS), we and others have demonstrated a diagnostic yield of approximately 25–35% depending on cohort characteristics [4, 7, 8]. Importantly, in these conditions a clinical test must capture the diverse landscape of disease-causing genetic variations, from single base pair alterations to chromosomal rearrangements. Small variants, including single nucleotide variants (SNVs) and insertions/deletions (INDELs) < 50 bp in size, are reliably detected by short-read genome [9, 10] and exome [8] sequencing. However, the detection of other variant types, such as short tandem repeat expansions (STRs), variants in paralogous regions and aberrant methylation, remains challenging. Furthermore, the complexity of structural variants (SVs) is often underestimated and balanced SVs are frequently missed by short read approaches [11–14]. Long-read GS refers to sequencing technologies capable of reading DNA fragments that are longer than those produced by short-read sequencers. Typically, short-read platforms generate reads of 150 base pairs. There is no clear definition of what a long read is, but they range from a few kilobases (kb) to megabases (Mb) of sequence. Currently, two commercially available platforms dominate the field: Oxford Nanopore Technologies (ONT) and Pacific Biosciences (PacBio). PacBio is known for its high sequencing accuracy, whereas ONT excels in generating longer reads. The systems also differ in throughput: PacBio’s current Revio platform can generate data equivalent to 4–6 human whole genomes per run, while ONT’s highest-capacity instrument can produce up to 48 human whole genomes per run. Both ONT and PacBio also detects epigenetic modifications including CpG methylation. Long-read is widely used in research, and has been shown to be a valuable tool in a diversity of tasks, including the creation of reference. Long-read is now emerging as a promising method in clinical genetic diagnostics, with the potential to detect the full spectrum of variant types that are often missed or incompletely resolved by short-read technologies. Early applications have shown that long-read GS is able to: (i) detect and accurately characterize SVs, even when they are complex or involve breakpoints in highly repetitive regions [15–17], (ii) precisely genotype pathogenic STR expansions [18], and (iii) identify disease-associated methylation patterns, both in the context of imprinted gene disorders and more broadly to aid the interpretations of variants of uncertain significance (VUS*)* [19]. At present, long-read GS remains more expensive than short-read approaches, largely due to the higher cost of flowcell reagents, and instruments, as well as bioinformatics development. However, costs are expected to decrease with increased throughput and automation which is expected to narrow the gap between the two technologies [20].

In this study we evaluated the utilization of long-read GS as a first line singleton clinical genetic test in children with neurological disorders. In a prospective, unselected cohort of 100 individuals referred to our laboratory for short-read GS, long-read GS achieved a comparable final diagnostic yield. Importantly, long-read GS provided a more comprehensive diagnostic workflow enabling direct phasing of compound heterozygous variants, detailed characterization of SVs and STRs and assessment of DNA methylation.

## Methods

Clinical genetics and Genomics (Karolinska University Hospital, Stockholm, Sweden) is a tertiary center where short-read GS (with in silico gene panel analysis) is performed as the first-line test for individuals with suspected rare genetic diseases, including NDDs, and malformation syndromes. Overall, roughly 5000 analyses are performed annually, the detailed analysis pipelines and overall workflow have previously been described [9].

In this study, we included 100 children and adolescents with neurological disorders referred to the Department of Clinical Genetics and Genomics (Karolinska University Hospital in Stockholm, Sweden), for diagnostic genetic testing with short-read GS between September 1, 2023, and April 15, 2024. The cohort consisted of 55 males and 45 females with a median age of five years and a phenotype suggestive of either a NDD (*n* = 79) or an NMD (*n* = 21). Participants were classified into the two subgroups according to the in silico gene panel selected based on the referral information. Informed consent was obtained either from the affected individuals or their legal guardians. To obtain a clinically representative first-line testing cohort, individuals were contacted directly after receiving the referral and before the short-read GS results were available. The first 100 individuals who gave consent and for whom DNA was available from both parents and the proband were included in the study; those without available parental or proband DNA were excluded. Although the study was conducted with singleton long-read GS, parental DNA was required for potential follow-up analyses. Clinical characteristics of the study cohort are summarized in Table 1.

**Table 1:** Cohort characteristics: Age, gender, and phenotypic presentation. The table presents age, gender, and the most common phenotypic traits reported in the referrals, organized according to human phenotype ontology (HPO) terms

COHORT CHARACTERISTICS	% (*N* = 100)
Median age, years (min-max)	5 (1 day − 20 years)
Male	55
Consanguinity	5
**Main phenotypic features (HPO terms)**	
Autism/Autistic behavior	56
Intellectual disability	36
Global developmental delay	21
Delayed speech and language development/Language impairment	21
Attention deficit hyperactivity disorder	10
Motor delay	6
Hypotonia	6
Microcephaly	6

Genomic DNA was extracted from whole blood. For the majority (*n* = 79), extraction was performed using QIAsymphony (QIAGEN, Hilden, Germany) and the QIAsymphony DSP DNA Midi Kit (QIAGEN, Hilden, Germany) according to the standard protocol. The other samples were extracted either by using QIAGEN EZ1 (*n* = 14) (QIAGEN, Hilden, Germany) or by manual extraction with the QIAamp DNA blood midi kit (*n* = 7) (QIAGEN, Hilden, Germany) according to manufacturer’s protocol.

### Short-read genome sequencing

Genomic DNA underwent library preparation using the Illumina TruSeq DNA PCR-free and was sequenced on a NovaSeqX (Illumina, San Diego, CA, USA). All samples were analyzed as singletons. The bioinformatic workflow calling SNV/INDELs, SVs, mobile element insertions, STR expansions as well as *SMN1*/*SMN2* copy number has been described previously [4, 9]. Based on phenotypic presentation, in-silico gene panels were applied: (i) 1568 genes associated with NDD and/or (ii) 1035 genes associated with NMD. In addition, STRs were assessed in hg19 in seven loci for NDD and in 32 for NMD (Additional file 1: Document S1). Detected variants were ranked and visualized as previously described [9, 21] and classified from 1 to 5 according to the American College of Medical Genetics and Genomics (ACMG)/Association for Molecular Pathology (AMP) guidelines [22]. In addition, a six-grade temperature scale of detected VUS was applied, ranging from hot to ice cold, in order of estimated pathogenicity [23]. The “hot VUSs” were reported to the referring physician as they were considered more likely to be disease-causing.

### Long-read sequencing

Genomic DNA was sheared with Covaris G-tube (17 samples; Covaris, Woburn, MA, USA) or Megaruptor (83 samples; Diagenode, Liège, Belgium) to obtain ~ 15–20 kb fragments followed by size selection using AMPure XP beads (Beckman Coulter, Brea, CA, USA) to remove fragments smaller than 2 kb. The libraries were prepared with the Ligation Sequencing Kit V14 and sequenced on a PromethION (Oxford Nanopore Technologies, Oxford, UK), using one PromethION R10.4.1 flow cell per sample. Wash and reload was performed after approximately 40 h of sequencing. For 12 samples, wash and reload was not performed as the number of active pores were well over 20% at the 40 h timepoint. Eight samples did not achieve enough data after the first run and they were re-sequenced on new flow cells. Raw data was processed through the custom pipeline Poorpipe [24]. In brief, the long-read GS data was aligned to reference genomes GRCh37 (hg19) and GRCh38 (hg38) using Minimap2 [25]. Variant calling was done using Claire3 (SNVs) [26], CNVpytor (CNVs) [27, 28], Sniffles (SVs) [29], methylartist (methylation) [30], Abacus (STRs) [31], and Paraphase (paralogous genes) [32]. The resulting data was phased using Whatshap [33], and annotated using Ensembl VEP [34]. The annotated variant lists were scored and ranked using Genmod [21]. Quality assessment was performed using Picard tools, samtools, Nanostats and bcftools, and the resulting reports were aggregated using MultiQC. A local database containing the long-read GS SV calls from the 100 cases was built for both hg19 and hg38 using SVDB [35]. In individual RD_P633 a *de novo* assembly was performed using HiFiasm [36]. The assembly was aligned to T2T-CHM13 using Minimap2 [25], and was inspected manually in Integrative Genomics Viewer (IGV) [37].

**Fig. 1:**
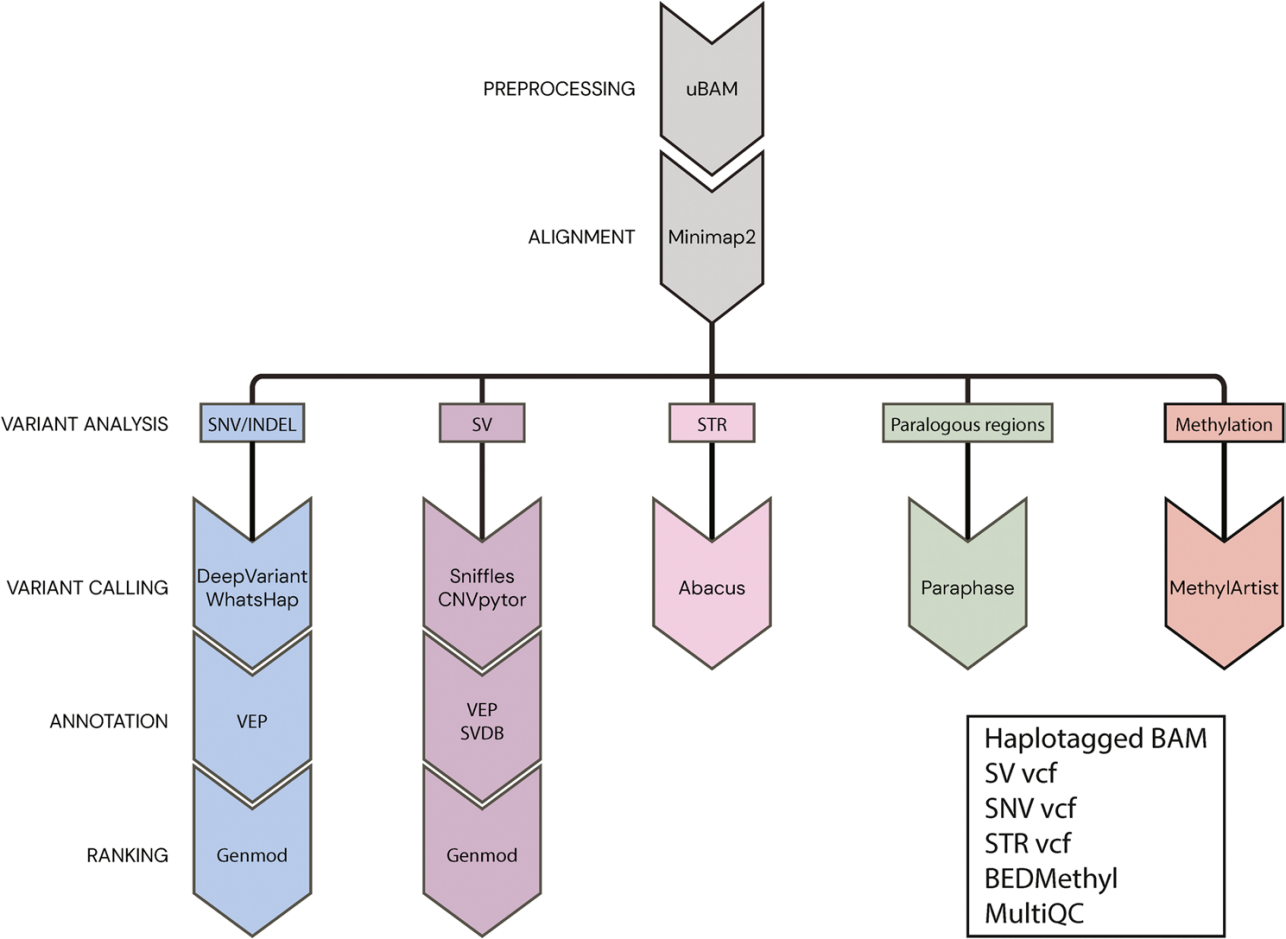
Flowchart of the long-read GS bioinformatic pipeline. Reads stored in binary alignment maps (BAMs) are aligned to the reference genome, followed by comprehensive variant calling, annotation and ranking. The variant callers include single nucleotide variants and small insertions/deletions (SNV/INDELs; blue), structural variants (SVs; purple), short tandem repeat (STR) expansions (pink), paralogous regions (green) as well as methylation calling (red)

The classification of detected variants was performed in the same way as for clinical short-read GS. SNVs, STR expansions in 52 loci (Additional file 1: Document S1) and paralogous regions (SMN) were assessed in our custom in-house interpretation software similar to the short-read GS data [9]. Imprinted regions were assessed using a custom script that compared the current case with a pool of controls (Additional file 1: Document S2). SVs were analyzed in two ways: large (> 10 kb) were assessed genome-wide and small (< 10 kb) were filtered based on gene panels. SVs localized within or in close proximity to genes included in the gene panel, as well as those involving more than two breakpoints, were examined in IGV [37] and the derivative chromosomes were resolved manually. Variants were interpreted clinically and classified in the same way as described for the short-read GS data. The long-read analysis pipeline is shown in Fig. 1.

### Orthogonal confirmation

Validation of the *FMR1* expansion was performed according to the manufacturer’s protocol, using the AmplideX PCR/CE *FMR1* kit (Asuragen, Austin, TX, USA) and an ABI 3500xL Genetic Analyzer (Applied Biosystems, Foster City, CA, USA).

To confirm aberrant methylation pattern across the Prader-Willi region, we performed methylation specific multiplex ligation-dependent probe amplification (MS-MLPA) using SALSA MLPA Probemix ME028 Prader-Willi/Angelman (MRC Holland, Amsterdam, Netherlands) according to manufacturer’s protocol.

## Results

### Genome data quality and characteristics

The long-read GS generated an average of 111 GB data per sample, with a median read-length of 5 kb and average N50 of 16 kb; resulting in an average coverage of 34X. Detailed quality and sequencing parameters are available in the Additional file 2: Table S1. The short-read GS achieved an average coverage of 33X and read length of 151 base pairs.

### Overall genetic findings

**Fig. 2:**
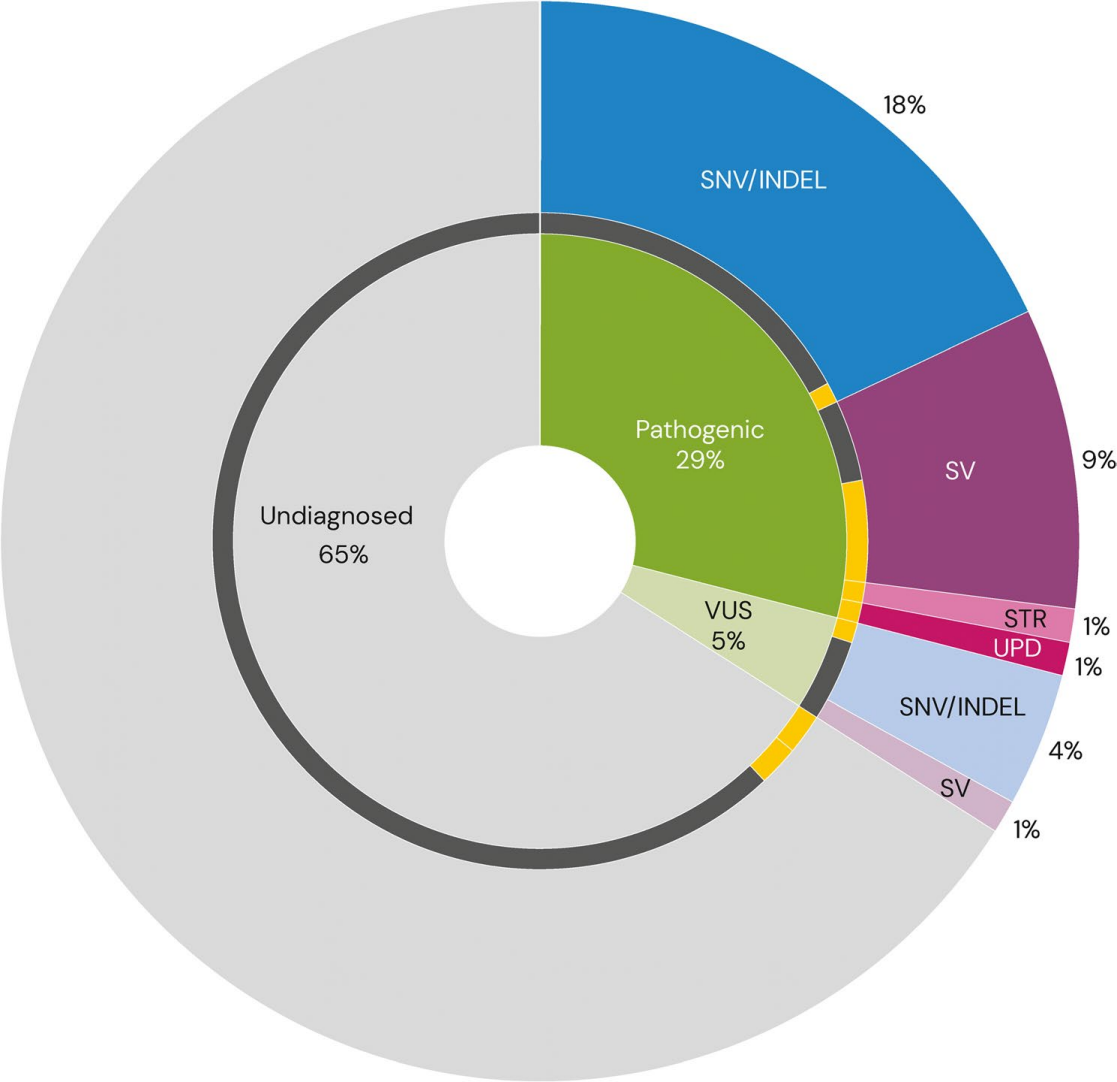
Diagnostic yield and added value of long-read genome sequencing (GS). Inner portion of pie chart illustrates the proportion of individuals with a pathogenic/likely pathogenic variant (dark green), individuals with a variant of uncertain significance (VUS) (light green), and undiagnosed individuals (grey). Outer portion depicts the types of variants identified: single nucleotide variants and/or small insertions/deletions (SNV/INDELs) (blue), structural variants (SVs) (purple), short tandem repeat (STR) expansion (pink) and uniparental disomy (UPD) (dark pink). The dark central circle represents the number of samples where long-read GS provided added clinical value (14%) (yellow)

In 100 unrelated individuals with neurodevelopmental (*n* = 79) or neuromuscular (*n* = 21) symptoms (Table 1), a likely genetic diagnosis was made in 29 individuals with pathogenic/likely pathogenic variants. A hot VUS was found in five additional individuals. In total, those individuals harbored 36 variants that were further subdivided into SNV/INDELs (*n* = 24), SVs (*n* = 10), STR (*n* = 1) and uniparental disomy (*n* = 1) (Fig. 2; Additional file 2: Table S2). In addition, five rare variants of clinical interest (three SVs and two STRs) were further investigated but finally classified as benign. All those 41 variants were detected with both short-read GS and long-read GS. The added value of long-read GS is showcased below for 13 cases from five different variant categories (SNV/INDELs, STRs, imprinted regions, paralogous regions and SVs).

### Added value of long-read GS

#### SNVs and indels

**Fig. 3:**
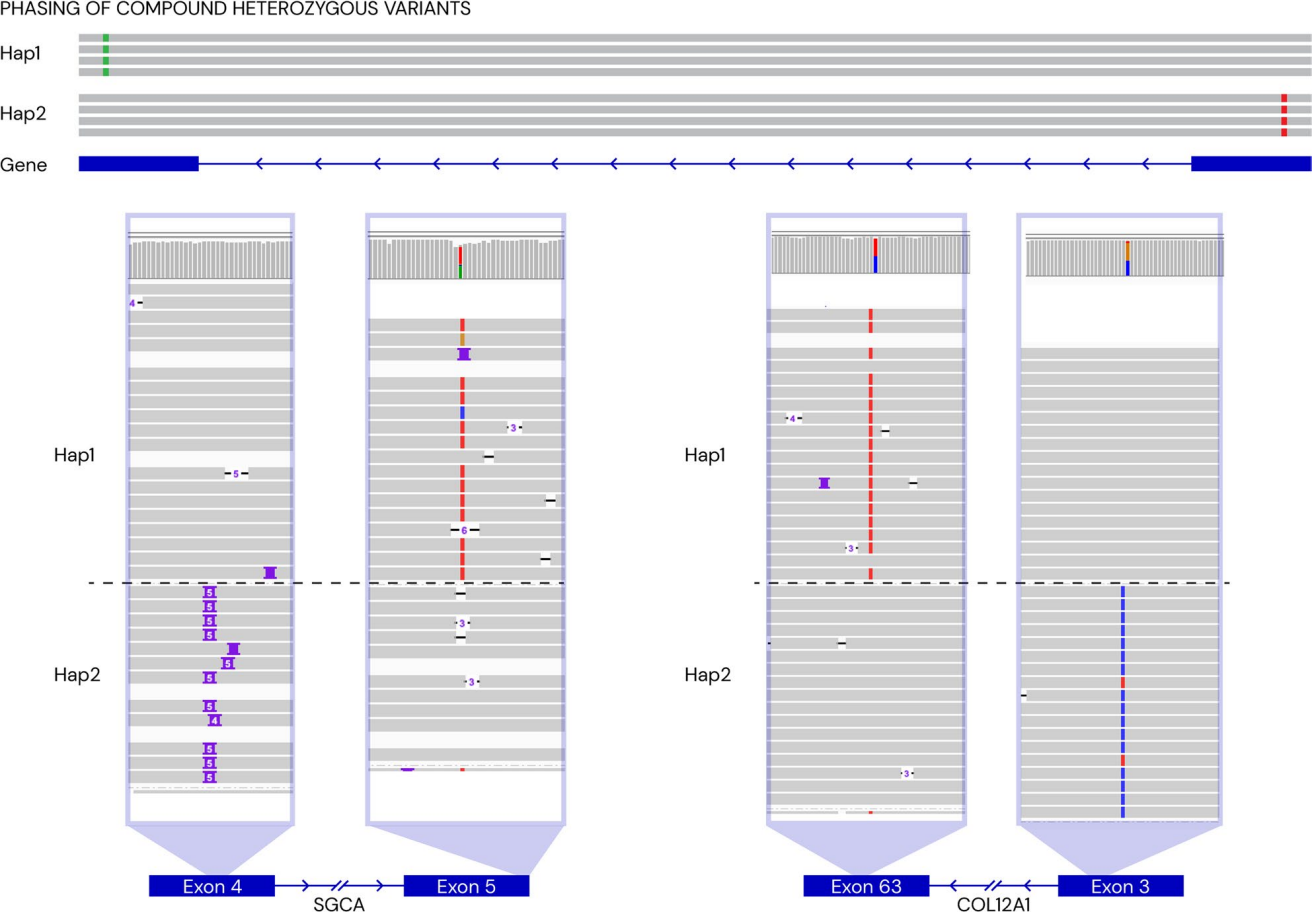
Phasing of compound heterozygous variants. Top, schematic illustration of long reads phased into haplotype 1 (Hap1) and haplotype 2 (Hap2), aligning across two exons separated by an intron. Hap1 carries a variant in exon 1 (green) and Hap 2 carries a variant in exon 2 (red). Bottom, phasing results from two individuals diagnosed with compound heterozygous variants: Left, an insertion of five bases in exon 4 of *SGCA* (Hap2) and a variant A > T in exon 5 (Hap1). Right, a variant G > A in exon 3 of *COL12A1* and a variant C > A in exon 63

Two individuals carried biallelic variants in two genes, *SGCA* (Limb-girdle muscular dystrophy, MIM# 608099) (RD_P694) and *COL12A1* (Bethlem myopathy 2, MIM# 616471) (RD_P712). In both, phasing the long-read GS data showed that the two variants were in *trans*, which was later confirmed by segregation analysis of parental samples (Fig. 3). This increased the support for pathogenicity for the variants in the *SGCA* gene that was upgraded to likely pathogenic, however the *COL12A1* variants remained as a hot VUS since the child was still very young and there was limited phenotypic information.

#### STR expansions

**Fig. 4:**
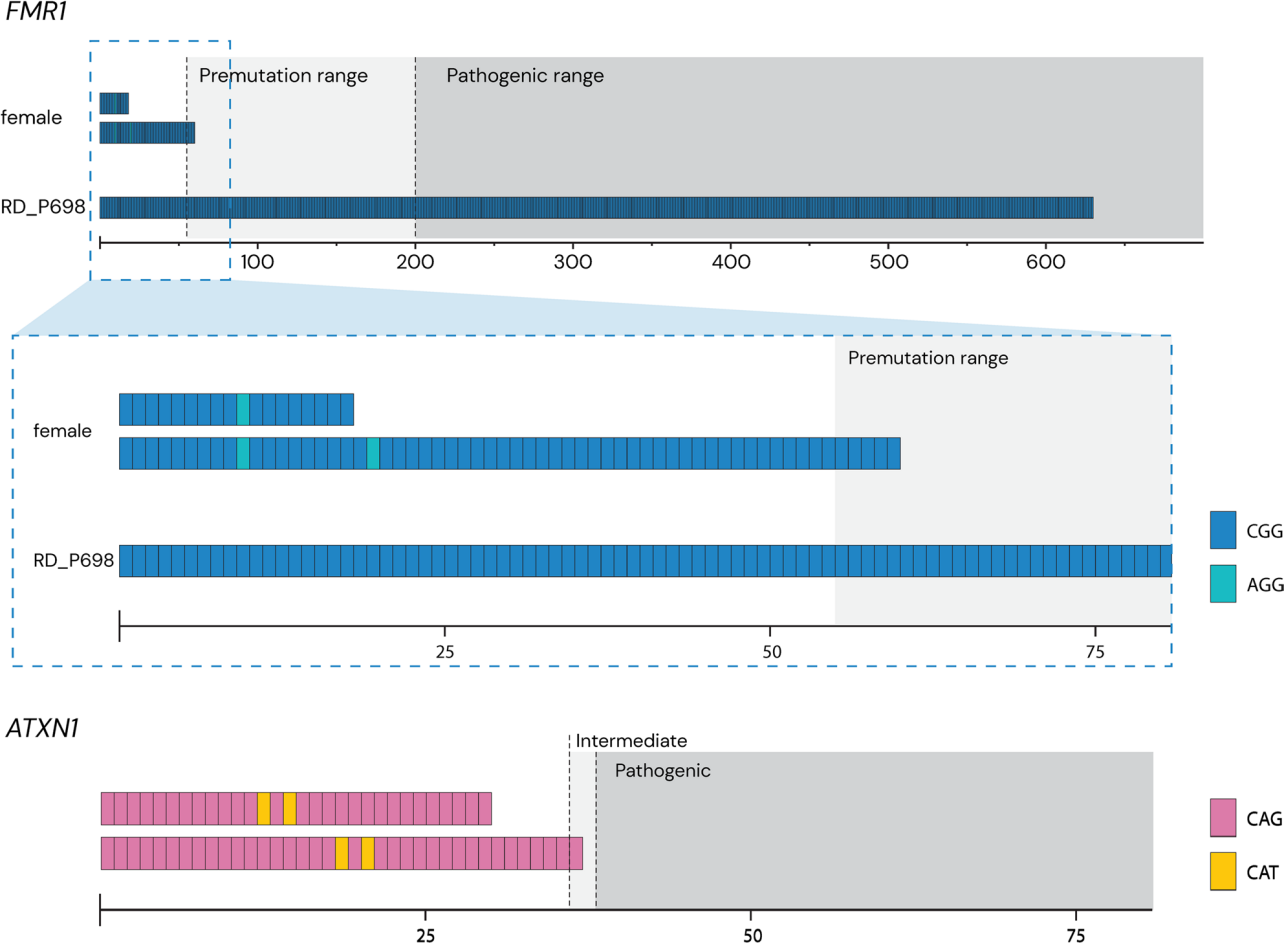
Illustration of short tandem repeat (STR) expansions. Top: STR analysis of a male (RD_P698) with full *FMR1* expansion of 654 CGG repeats, a female with an premutation-range expansion of 60 repeats in *FMR1*. The zoom-in highlights the presence of interrupting motifs in the female, and the loss of interrupting motifs in the boy. Bottom: STR analysis of *ATXN1* in an individual with one alleles in the intermediate range (37 repeats) with two interrupting CAT motifs

A pathogenic *FMR1* expansion (Fragile X syndrome, MIM# 300624) was detected in a two-year-old male with global developmental delay (RD_P698). With long-read GS the CGG repeat size was found to have a median length of 654, with short-read GS 76 and targeted *FMR1* testing showed > 200. Although the repeat size was estimated at 654 units, the STR caller identified variable length of the expansion with repeat lengths ranging between 375 and 700 repeats respectively (Fig. 4, Additional file 3: Fig. S1). Methylation analysis further revealed that the *FMR1* promoter was hypermethylated (Additional file 3: Fig. S1). This promoter hypermethylation is associated with gene silencing and underlies the pathogenic effect of full *FMR1* expansions. In comparison, in another patient, long-read GS also detected an *FMR1* allele with 60 repeats and two interrupting AGG motifs that was not hypermethylated, but predominantly unmethylated (Additional file 3: Fig. S1). A finding not interpreted as the cause of clinical symptoms. Finally, for an *ATXN1* allele with 37 repeats, long-read GS allowed haplotype-resolved sizing (31 and 37 repeats) and revealed two interrupting CAT motifs in each allele (Fig. 4). Since interrupting CAT motifs in allele sizes spanning 36–44 repeats are considered normal, the variant could be dismissed.

#### Imprinting disorders

In a newborn boy (RD_P706) with neonatal hypotonia, long-read GS detected partial maternal uniparental isodisomy of chromosome 15. Because this isodisomy was in proximity to the Prader-Willi region, it raised the suspicion of uniparental heterodisomy and prompted us to perform methylation analysis. Methylation analysis revealed hypermethylated promoter regions of multiple genes in the Prader-Willi region, including *NDN* (z-score − 6.49), *SNRPN* (z-score − 3.35) and *MAGEL2* (z-score − 13.5) that could also be visually inspected in IGV (Additional file 1: Document 2; Additional file 3: Fig. S2). Although short-read GS also captured the uniparental disomy, it required orthogonal confirmation using methylation specific multiplex ligation-dependent probe amplification (MS-MLPA) to confirm the presence of aberrant methylation patterns associated with Prader-Willi syndrome (MIM#176270) (Additional file 3: Fig. S3-S4).

#### Paralogous regions

A three-year-old girl with progressive muscle weakness (RD_P623) had a homozygous deletion of *SMN1* and four copies of *SMN2* consistent with spinal muscular atrophy type 3 (MIM# 253400). While the copy number of *SMN1* and *SMN2* were detected by short-read GS using the analysis software SMNCopyNumberCalling [38], MLPA was needed for confirmation. In contrast, phased long-read GS data could directly detect the homozygous deletion of *SMN1* and distinguish the four *SMN2* copies (Additional file 3: Fig. S5).

#### Structural variants

Including the above mentioned *SMN1* deletion, a total of 14 rare potentially clinically significant SVs were detected (Additional file 2: Table S2) and the long-read GS data enabled a complete characterization in all of them; seven representative cases are highlighted below, including both simple SVs and complex events.

#### Simple SVs

In three simple SVs, identified in two individuals, long-read GS aided in the interpretation. First, in a 10-year-old male with intellectual disability (RD_P633), long-read GS was not only able to confirm the short-read call of a terminal duplication on chromosome 8, dup(8)(p23.3p23.1), but also identified telomeric sequence at one of the breakpoints and a link to the acrocentric p-arm of chromosome 14 at the other breakpoint indicating an unbalanced translocation. However, T2T-CHM13 alignment was required to fully resolve the translocation. Second, in a one-year-old boy (RD_P695) with delayed motor development and poor balance, two inversions were readily identified by long-read GS. One pathogenic event, an inversion on chromosome 13, inv(13)(q31.1q33.1), disrupts *FGF14* resulting in the diagnosis of Spinocerebellar ataxia 27 A, SCA27A, MIM#193003). The other event was a 23 Mb pericentric inversion on chromosome 10, inv(10)(p11.21;q21.1). The pathogenic event was observed in the clinical short-read GS analysis, but not the inv(10). However, manual inspection of the VCF files showed that the inv(10) was also called in short-read GS data, but the event was not highly ranked. Of note, in a one-year-old girl with global developmental delay, a duplication of 22q11 was detected from read depth analysis, although characterization of the breakpoints was not possible.

#### Complex SVs

**Fig. 5:**
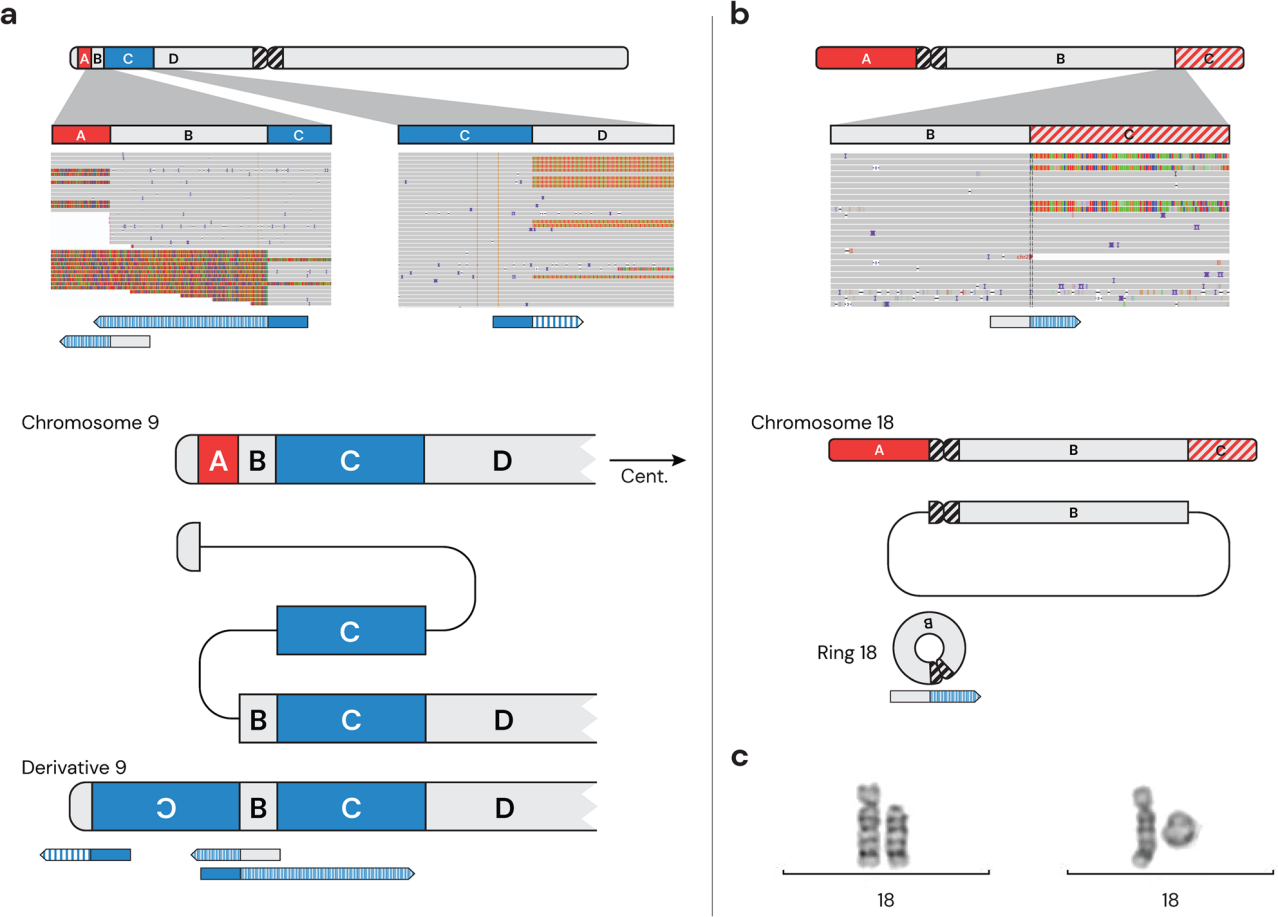
Resolved structure of of complex structural variants (SVs). **a** DEL-INV-DUP rearrangement of chromosome 9. Top: Schematic showing the deleted (A) and duplicated (C) segments of chromosome 9p, with integrative genomics viewer (IGV) screenshots of the breakpoint regions. Below, an illustration of soft-clipped reads at the breakpoints. Bottom: An illustration of the reference chromosome 9, a subway plot of the rearrangement, and the resolved derivative structure. Below this, the alignment of soft-clipped reads on the derivative. **b** Mosaic ring chromosome 18. Top: Schematic showing a deletion 18p (A) and mosaic deletion on 18q (C), with IGV screenshots of the breakpoint region at 18q. Below, an illustration of a soft-clipped read at the breakpoint. Bottom: Illustration of the reference chromosome 18, a subway plot of the ring chromosome, and the resolved derivative structure. Below this, alignment of the soft-clipped read on the derivative. **c** Karyotyping images showing deletion of 18p (left) and ring chromosome 18 (right)

Five complex SVs in four individuals were resolved with long-read GS (Table 2). A DEL-INV-DUP rearrangement on chromosome 9 was found in a two-year-old girl (RD_P651) with syndromic global developmental delay. The identified structural rearrangement consisted of a terminal 1.8 Mb deletion at 9p24.3 and a 17.8 Mb duplicated segment at 9p24.3p22.1, separated by an 850-bp normal copy number segment. The duplicated segment had replaced the deleted segment, and was inserted in an inverted orientation, so that the normal copy number segment was flanked by two mirroring identical segments (Fig. 5a). Long-read GS could fully resolve the derivative chromosome structure and although short-read GS detected the CNVs involved, no call (in the clinical interpretation tool) allowed us to resolve the structure of the rearrangement. In a 9-month-old girl RD_P655 with motor delay, long-read GS identified a heterozygous deletion of chromosome 18p and a mosaic deletion of the distal 18q. Six out of 28 long-read GS reads connected the q-arm to the p-arm indicating a mosaic ring chromosome in 42% of the cells (Fig. 5b). The ring chromosome was also detected by karyotyping; 46,XX, del(18)(p11.1) [31]/46,XX, r(18)(p11.1q23) [8]. Re-evaluation of the ring breakpoint junction in IGV, found that it was not visible in the short-read data. In a one-year-old girl (RD_P649) with global developmental delay a complex rearrangement of chromosome 1 was resolved with long-read GS. The five duplicated segments were inserted into chromosome 1 in a seemingly unorganized manner. Although three genes overlapped with the breakpoints, all retain a remaining functional copy (Additional file 3: Fig. S6). The complex SV was inherited from the unaffected mother and classified as likely benign, while a pathogenic *de novo* SNV/INDEL in *ARID1A* (c.3236-3239delinsG) led to a clinical diagnosis of Coffin-Siris syndrome 2 (MIM#614607). In a 13-year-old boy with intellectual disability (RD_P658), two separate complex SVs, a DUP-TRP/INV-DUP on chromosome 9 and a DEL-DUP on chromosome 10, were resolved by long-read GS (Additional file 34: Fig. S7). Parental analysis showed that the two events were inherited from the same healthy parent, and both were classified as benign. The affected CNVs were also identified with short-read GS but the rearrangements could not be phased.

**Table 2:** Overview of 17 variants with added clinical value from long-read genome sequencing (GS). The table summarizes 17 variants identified in 13 individuals, grouped by variant type: single nucleotide variant (SNV) and/or small insertion/deletion (INDEL), structural variant (SV), short tandem repeat (STR) expansion, and other. Identifying information has been removed for variants that were not reported (NR) to avoid disclosing non-reportable findings

Individual	Age	Sex	Affected gene	Variant/s	Added value of long-read GS	Outcome
**Single nucleotide variants and/or small insertion/deletion**						
RD_P694	6 y	F	*SGCA* (NM_000023.4)	c.557 A > T, p.Lys193Ter c.348_352dup, p.Gln118ProfsTer95	The SNV and INDEL), 585 bp apart, were phased in *trans*	LP
RD_P712	1 d	F	*COL12A1* (NM_004370.6)	c.8861G > A p.Gly2954Glu c.165 C > G p.Tyr55Ter	The two SNVs, 105 kb apart, were phased in *trans*	Hot VUS
**Structural variant (SV)**						
RD_P623	3 y	F	*SMN1*	Homozygous deletion of *SMN1*	Phasing of paralogous regions, detecting no copy of *SMN1* and phasing four copies of *SMN2*	P
RD_P695	1 y	M	*FGF14*	Inversion of 13q disrupting *FGF14* Inversion of chr 10	Mapping of SV breakpoints and identification of a 7.6 kb deletion Detection of a pericentric inversion of chromosome 10	P LB
RD_P633	10 y	M	multiple	Unbalanced translocation der(14)t(8;14)	Detected telomeric sequence at one breakpoint Detected link to acrocentric p-arm at the other breakpoint	P
RD_P651	2 y	F	multiple	Complex rearrangement of 9p	Resolved CGR structure	P
RD_P655	9 m	F	multiple	Mosaic ring chromosome 18/deletion of 18p	Resolved CGR structure	P
NA	-	-	none	Complex SV involving 9p Complex SV involving 10p	Calling of SV and mapping of breakpoints that did not disrupt disease-causing genes	LB (NR)
NA	-	-	none	Complex SV involving 1p	Calling of SV and mapping of breakpoints that did not disrupt disease-causing genes	LB (NR)
Short tandem repeat (STR) expansion*						
RD_P698	2 y	M	*FMR1*	Full expansion (654 CGG repeat)	Characterization of STR expansion; size, loss of interrupting motifs, hypermethylation	P
NA	-	F	*FMR1*	Expansion (60 CGG repeat)	Characterization of STR and detection of interrupting motifs	LB (NR)
NA	-	-	*ATXN1*	Expansion (37 CAG repeat)	Characterization of STR and detection of interrupting motifs	LB (NR)
**Other**						
RD_P706	11 d	M	multiple	Maternal uniparental isodisomy of chromosome 15	Methylation analysis confirms PWS diagnosis	P

*NA* not applicable, *y* year, *m* month, *d* day, *F* female, *M* Male, *chr* chromosome, *bp* base pair, *CGR* complex genomic rearrangement, *PWS* Prader-Willi syndrome, *LB* likely benign, *LP* likely pathogenic, *P* pathogenic, *VUS* variant of uncertain significance. * median length

#### Orthogonal confirmation, parental follow-up and sequential testing

In the clinical short-read GS workflow, 39 targeted tests of probands and parental follow-up for 42 probands resulted in a 9% and 20% increase in cost, respectively (Additional file 2: Table S3.

–S5). This process led to the reclassification of 19 variants: six were upgraded to pathogenic/likely pathogenic and 13 were downgraded to likely benign. The remaining 23 variants retained their original classification, 18 of which had already been considered pathogenic/likely pathogenic. It is estimated that 24% (20/83) of parental tests and all targeted tests of probands could be omitted as part of the diagnostic process if long-read GS was applied.

#### SVs and STRs in hg 38

In total, we identified 34,603 and 33,863 SVs in the long-read GS data when aligned to hg19 and hg38 respectively. The hg38-aligned SVs were further processed by (i) filtering for unique events using our internal database and (ii) assessing overlap with genes included in curated gene lists. STRs were assessed at 52 loci. However, neither analysis revealed any clinically relevant findings.

## Discussion

The diverse genetic background observed in neurological disorders such as NDDs and NMDs prompted us to evaluate the clinical value of singleton long-read GS as a first line genetic test in this patient group. Although long-read GS did not increase the overall yield it provided clinically relevant information such as phasing, characterization of STR expansions, and resolution of SV structures, resulting in improved variant assessment compared to short-read GS. In addition, methylation analysis shows strong promise as a clinically valuable layer of information.

Short-read GS has been a major success in rare disease diagnostics, enabling a streamlined workflow for individuals with suspected genetic conditions. Nevertheless, a substantial proportion of cases remain undiagnosed, with overall diagnostic yields of 25–50% depending on inclusion criteria [9, 10]. While some of these cases may have non-genetic causes or involve as-yet undiscovered disease mechanisms, such as pathogenic non-coding variants or complex inheritance patterns including digenic or oligogenic transmission, the inherent limitations of the short-read technologies also contribute to the reduced detection rates. These limitations include missed or incorrectly resolved SV breakpoints in repetitive regions and poor genotyping accuracy of both SVs and STRs [14, 39]. Furthermore, phasing is generally not possible, making it difficult to distinguish whether a variant is located in a gene of interest or its pseudogene. In addition, short reads cannot span across multiple variants in the same gene, especially when they are located in different exons. A clear and critical step likely to improve rare disease diagnostics is the implementation of long-read GS, which promises a more comprehensive variant characterization and a simpler interpretation process compared to short-read GS [40–42]. Recent studies have suggested a potential increase in diagnostic yield of up to 7.3% with long-read GS [42]. In this study, we focused on individuals with neurologic disorders with onset during childhood, a group known to have a genetically heterogeneous background.

The current study highlights the advantages of long-read GS in clinical diagnostics. We observed a benefit in 13 individuals: two with biallelic variants in autosomal recessive disease genes, seven harboring nine resolved SVs, three with STR expansions, and one with UPD (Table 2). For SNVs, the main benefit of long-read GS was phasing; for example, directly confirming that the two variants detected in *SGCA* (RD_P694) were in trans without requiring parental follow-up. The ability to phase was also important in paralogous regions which was demonstrated by the identification of a homozygous deletion of SMN1 in RD_P623 together with the accurate determination of four SMN2 copies (Table 2, Additional file 3: Fig. S5). Comprehensive characterization of STR expansions, including the identification of a pathogenic *FMR1* expansion in RD_P698 with CGG repeat sizes ranging from ~ 354 to ~ 680 units (Fig. 4; Table 2, Additional file 3: Fig. S1). The variability of repeat counts between molecules may reflect mitotic instability or technical variability inherent to long-read sequencing [43].

The most important contribution to the clinical analysis was improved SV calling, which provided the greatest added value and is likely to be one of the main benefits of long-read GS. Many of the identified SVs had breakpoints in regions that are typically inaccessible to short reads, including pericentromeric, telomeric, and acrocentric p-arms. Examples include the complex DEL-INV-DUP rearrangement on chromosome 9 (RD_P651), a mosaic ring chromosome 18 (RD_P655), and an unbalanced translocation der(14)t(8;14) (RD_P633), all of which were resolved in detail by long-read GS. These cases illustrate how long-read data can elucidate structural events that were previously only approachable through karyotyping or other labor-intensive cytogenetic work. Nonetheless, recurrent copy number variants flanked by large segmental duplications, such as dup(22)(q11) (RD_P630), remain challenging. Here, breakpoints could not be fully resolved due to short DNA fragments, underlining the importance of high-quality input material and the continued need for more complete reference assemblies. Finally, the reference remain a limiting factor highlighted by need of T2T-CHM13 to resolve the above-mentioned unbalanced translocation.

Long-read based methylation analysis is a highly promising clinical tool both for verification of genetic variants and as functional readout of the variants’ effects. In our cohort we detected two straightforward clinically relevant methylation use cases. First, long-read GS detected both the genetic and epigenetic abnormalities, establishing a diagnosis of Prader-Willi syndrome in a single experiment (RD_P706), whereas short-read GS only detected the isodisomy and MS-MLPA was needed to verify the diagnosis. Second, long-read GS could detect the methylation status of the *FMR1* promoter confirming pathogenicity of the detected STR expansions identified in individual RD_P698. Analysis of global methylation patterns was not applied in our study, although there appears to be a strong potential for its diagnostic utility [44]. To fully harness the potential of methylation calling, large reference methylation databases need to be established based on long-read GS data.

A key strength of this study is the use of a representative cohort, consisting of individuals referred for their first genomic analysis, which allowed us to evaluate long-read GS as a true first-line diagnostic test. Another important aspect is the use of routine DNA extractions, compatible with standard clinical laboratory workflows. This meant no changes were required in sample collection, extraction, or storage protocols, enabling straightforward integration into existing diagnostic pipelines. Despite the relatively modest read lengths obtained from regular DNA, long-read GS delivered robust performance and added clinical value across multiple variant types.

A limitation of this study is that long-read GS data were analyzed against the hg19 reference genome, reflecting the current standard in our short-read clinical workflow [9]. This ensured unbiased comparison between technologies, but it also constrained detection in regions that are better represented in GRCh38, such as centromeres and telomeres [45]. More complete assemblies, including T2T-CHM13, enable more accurate mapping in highly repetitive regions and have proven critical for resolving certain clinically relevant structural variants [11, 12, 16, 17, 41]. Future analyses aligned to GRCh38 and T2T-CHM13, ideally combined with de novo assembly, will further improve SV detection and characterization. Another limitation is that we were not able to perform a global analysis of methylation patterns across the cohort as discussed above. Finally, the DNA quality may have influenced the read lengths obtained in this study. The use of routine DNA extractions enabled seamless integration into clinical workflows but resulted in relatively short read lengths (~ 5 kb on average). Although this was sufficient for phasing variants and resolving many SVs, longer DNA fragments would improve resolution in regions flanked by large segmental duplications and facilitate more comprehensive genome-wide analyses.

The current flowcell and reagent prices of long-read GS is approximately three times those for short-read GS (Additional file 2: Table S3). In addition, the technology is still highly hands-on and less scalable than short-read GS, for which automated solutions enable high throughput production. Hence, costs will likely remain higher for the foreseeable future. However, these higher per-sample costs need to be balanced against the ability to provide a comprehensive genetic diagnosis in a single test, thereby reducing the need for multiple sequential investigations, both shortening the time to diagnosis and streamlining the clinical diagnostic workflow. In contrast, short-read GS often requires additional targeted testing to capture variants not fully resolved (e.g., *SMN1*, STR expansions, complex SVs, and methylation changes) as well as segregation testing of variants in autosomal recessive genes. While parental samples remain important for recurrence risk assessment, some of these costs could be reduced when long-read GS is used as a first-line test. In this study, long-read GS would have reduced the need for additional tests by approximately 50%; however, the exact cost savings will depend on local pricing structures, reimbursement policies, and laboratory workflows. Although turnaround time was not formally measured in this study, several aspects can be anticipated. Interpretation of long-read GS may initially take longer than short-read GS, as it captures a greater number of variants and fewer are filtered out by population frequency due to the currently smaller local reference database (> 100 samples compared with > 30,000 for short-read GS at our center). However, when both local and public databases expand, interpretation time is expected to decrease and may ultimately become shorter than for short-read GS. In the long term, this streamlined approach is likely to reduce the need for confirmatory testing, shorten diagnostic turnaround times, and provide a clear health-economic advantage.

## Conclusions

This study demonstrates that singleton long-read GS is a powerful first-line diagnostic tool for neurological disorders, enabling comprehensive detection of SNVs, SVs, STR expansions, and phasing even from routine clinical DNA. We show that this technology can resolve clinically relevant variants in repetitive and previously inaccessible genomic regions, providing insights beyond the reach of short-read GS. The ability to simultaneously assess epigenetic changes, such as methylation, adds further diagnostic value, particularly for imprinting disorders. While the overall diagnostic yield was equal to short-read GS in this cohort, long-read GS offered superior resolution and interpretive power, reducing the need for follow-up testing and enabling more precise variant characterization.

Looking ahead, broader implementation of long-read GS promises to transform clinical diagnostics. As sequencing throughput increases, costs decrease, and laboratory automation improves, long-read GS is moving closer to becoming an affordable and scalable first-tier test. To support this transition, further research is needed to benchmark performance in larger and more diverse cohorts, develop optimized bioinformatic tools, and integrate more complete reference genomes such as T2T-CHM13. Building clinical infrastructure and generating robust health-economic evidence will also be critical to demonstrate feasibility at scale. With these advances, long-read GS has the potential to deliver more comprehensive and accurate diagnoses, improve clinical decision-making, and accelerate precision medicine for individuals with rare diseases.

## Supplementary Information

Supplementary Material 1.

Supplementary Material 2.

Supplementary Material 3.

## Data Availability

Code for Poorpipe is available at [https://github.com/J35P312/poorpipe](https://github.com/J35P312/poorpipe) and [10.5281/zenodo.17977821](10.5281/zenodo.17977821). The datasets discussed in this article are not immediately accessible due to ethical and privacy constraints but are available from the corresponding author on reasonable request. The data will ultimately be made available through the European ‘1+ Million Genomes’ Initiative and the Federated European Genome-phenome Archive (FEGA), which is currently being established. The Swedish national node, FEGA Sweden, enables controlled-access data sharing. Datasets deposited in FEGA Sweden will be findable via the European Genome-phenome Archive web portal ([https://ega-archive.org](https://ega-archive.org)). Clinical case data will be stored at the National Genomics Platform managed by Genomic Medicine Sweden (accession: GMS-RD_00003).
