# Supplementary material for: Long-read genome sequencing enhances diagnostics of pediatric neurological disorders
Source: Genome Med. 2026 Jan 9;18:12. doi: 10.1186/s13073-025-01596-5 (PMC12838436; doi:10.1186/s13073-025-01596-5)
Supplement: Supplementary file 1 — Supplementary Material 1. [file 13073_2025_1596_MOESM1_ESM.pdf]

Document S1: List of genes in *in-silico* gene panels for intellectual disability and neuromuscular disorder.

Panel of 1568 genes associated with intellectual disability

RNU4-2, CNBP, AAAS, AARS1, ABAT, ABCA2, ABCC9, ABCD1, ABCD4, ABHD16A, ABHD5, ACAD9, ACADM, ACBD6, ACO2, ACOX1, ACSL4, ACTB, ACTG1, ACTL6A, ACTL6B, ACY1, ADAM22, ADAMTS10, ADAR, ADARB1, ADAT3, ADD1, ADD3, ADGRG1, ADGRL1, ADK, ADNP, ADSL, AFF2, AFF3, AFF4, AGA, AGMO, AGO1, AGO2, AGTPBP1, AHCY, AHDC1, AHI1, AIFM1, AIMP1, AKT3, ALDH18A1, ALDH3A2, ALDH4A1, ALDH5A1, ALDH7A1, ALG1, ALG11, ALG12, ALG13, ALG14, ALG3, ALG6, ALG8, ALG9, ALKBH8, ALMS1, AMER1, AMOTL1, AMPD2, AMT, ANK2, ANK3, ANKRD11, ANKRD17, AP1B1, AP1G1, AP1S1, AP1S2, AP2M1, AP3B1, AP3B2, AP4B1, AP4E1, AP4M1, AP4S1, APC2, COA8, ARCN1, ARF1, ARF3, ARFGEF1, ARFGEF2, ARG1, ARHGAP35, ARHGEF9, ARID1A, ARID1B, ARID2, ARL13B, ARL6, ARMC9, ARPC4, ARSA, ARSB, ARSL, ARV1, ARX, ASAH1, ASH1L, ASL, ASNS, ASPA, ASPM, ASS1, ASTN1, ASXL1, ASXL2, ASXL3, ATAD1, ATAD3A, ATG4D, ATG7, ATIC, ATN1, ATP13A2, ATP1A1, ATP1A2, ATP1A3, ATP2B1, ATP5PO, ATP6AP1, ATP6AP2, ATP6V0A1, ATP6V0A2, ATP6V0C, ATP6V1A, ATP6V1B2, ATP7A, ATP8A2, ATP9A, ATR, ATRX, AUH, AUTS2, B3GALNT2, B3GLCT, B4GALNT1, B4GALT1, B4GALT7, B9D2, BAP1, BAZ2B, BBS1, BBS10, BBS12, BBS2, BBS4, BBS5, BBS7, BBS9, BCAP31, BCAS3, BCKDHA, BCKDHB, BCKDK, BCL11A, BCL11B, BCOR, BCORL1, BCS1L, BICRA, BLOC1S1, BMP4, BOLA3, BPTF, BRAF, BRAT1, BRD4, BRF1, BRPF1, BRSK2, BRWD3, BSCL2, BSND, BTD, BUB1B, C12orf4, C12orf57, MTRFR, VPS35L, C2CD3, C2orf69, CPLANE1, CA2, CA8, CACNA1A, CACNA1B, CACNA1C, CACNA1D, CACNA1E, CACNA1G, CACNA1I, CACNA2D1, CACNA2D2, CAD, CAMK2A, CAMK2B, CAMK4, CAMSAP1, CAMTA1, CAPN15, CAPRIN1, CARS1, CARS2, CASK, CBL, CBS, CBX1, CBY1, CC2D1A, CC2D2A, CCBE1, CCDC115, CCDC22, CCDC47, CCDC82, CCDC88A, CCDC88C, CCND2, CDC42, CDC42BPB, CDH11, CDH2, CDK10, CDK13, CDK16, CDK19, CDK5RAP2, CDK8, CDKL5, CDON, CELF2, CENPF, CENPJ, CEP104, CEP120, CEP135, CEP152, CEP164, CEP290, CEP41, CEP55, CEP57, CEP83, CEP85L, CHAMP1, CHD1, CHD2, CHD3, CHD4, CHD5, CHD7, CHD8, CHKA, CHKB, CHMP1A, CIC, CIT, CKAP2L, CLCN3, CLCN4, CLCN6, CLCNKB, CLDN5, CLN3, CLN5, CLN6, CLN8, CLP1, CLPB, CLTC, CNKSR2, CNNM2, CNOT1, CNOT2, CNOT3, CNOT9, CNPY3, CNTNAP1, CNTNAP2, COASY, COG1, COG4, COG5, COG6, COG7, COG8, COL4A1, COL4A2, CERT1, COLEC11, COPB2, COQ4, COQ8A, COX10, COX11, COX15, CPE, CPS1, CPSF3, CRADD, CRB2, CREBBP, CRIPT, CRLS1, CSDE1, CSNK1G1, CSNK2A1, CSNK2B, CSPP1, CTBP1, CTCF, CTDP1, CTNNA2, CTNNB1, CTR9, CTSA, CTSD, CTU2, CUL3, CUL4B, CUX1, CUX2, CWC27, CWF19L1, STEEP1, CYB5R3, CYFIP2, D2HGDH, DAG1, DARS1, DARS2, DBT, DCAF17, DCHS1, DCPS, DCX, DDB1, DDC, DDHD2, DDX11, DDX23, DDX3X, DDX59, DDX6, DEAF1, DEGS1, DENND5A, DENND5B, DEPDC5, DHCR24, DHCR7, DHDDS, DHFR, DHPS, DHTKD1, DHX30, DHX37, DHX9, DIAPH1, DIS3L2, DKC1, DLD, DLG3, DLG4, DLL1, DMAP1, DMD, DMXL2, DNAJC12, DNAJC19, DNM1, DNM1L, DNMT3A, DNMT3B, DOCK3, DOCK6, DOCK7, DOHH, DOLK, DPAGT1, DPF2, DPH1, DPH5, DPM1, DPM2, DPYD, DPYS, DPYSL5, DRG1, DTYMK, DYM, DYNC1H1, DYNC1I2, DYRK1A, EARS2, EBF3, EBP, EDEM3, EED, EEF1A2, EEF1B2, EEF2, EFTUD2, EHMT1, EIF2AK2,

EIF2AK3, EIF2S3, EIF3F, EIF4A2, EIF4A3, EIF5A, ELAC2, ELOVL4, ELP2, EMC1, EMC10, EML1, ENTPD1, EP300, EPG5, EPRS1, ERCC1, ERCC2, ERCC3, ERCC5, ERCC6, ERCC6L2, ERCC8, ERI1, ERLIN2, ESAM, ESCO2, ETFA, ETFB, ETFDH, ETHE1, EXOC7, EXOSC3, EXOSC8, EXT2, EXTL3, EZH1, EZH2, HYCC1, FAM149B1, FAM20C, FAM50A, FAR1, FARS2, FARSA, FARSB, FASTKD2, FAT4, FBRSL1, FBXL3, FBXL4, FBXO11, FBXO28, FBXO31, FBXW11, FBXW7, FDFT1, FEM1C, FGD1, FGF12, FGF13, FGF14, FGFR1, FGFR3, FH, FIG4, FITM2, FKRP, FKTN, FLNA, FLVCR2, FMN2, FMR1, FOLR1, FOSL2, FOXG1, FOXP1, FOXP2, FOXRED1, FRA10AC1, FRAS1, FRMD5, FRMPD4, FRRS1L, FSD1L, FTCD, FTO, FTSJ1, FUCA1, FUT8, FZR1, GABBR1, GABBR2, GABRA1, GABRA2, GABRA3, GABRA5, GABRB2, GABRB3, GABRD, GABRG2, GAD1, GALC, GALE, GALNT2, GALT, GAMT, GATA6, GATAD2A, GATAD2B, GATM, GCDH, GCH1, GCSH, GDI1, GEMIN4, GEMIN5, GFAP, GFER, GFM1, GFM2, GIGYF1, GJC2, GK, GLB1, GLDC, GLI2, GLI3, GLIS3, GLRA2, GLS, GLUL, GLYCTK, GM2A, GMPPA, GMPPB, GNAI1, GNAO1, GNAS, GNB1, GNB2, GNB5, GNE, GNPAT, GNPTAB, GNPTG, GNS, GOLGA2, GOT2, GPAA1, GPATCH11, GPC3, GPC4, GPRC5B, GPT2, GRIA1, GRIA2, GRIA3, GRIA4, GRID2, GRIK2, GRIN1, GRIN2A, GRIN2B, GRIN2D, GRM1, GRM7, GSS, GTF2H5, GTF3C3, GTPBP2, GTPBP3, GUSB, H3-3A, H3-3B, HACE1, HADHA, HADHB, HCCS, HCFC1, HCN1, HDAC2, HDAC4, HDAC8, HECTD4, HECW2, HEPACAM, HERC1, HERC2, HESX1, HEXA, HEXB, HGSNAT, HIBCH, HID1, HIKESHI, HIRA, H1-4, H4C3, H4C5, H4C9, HIVEP2, HK1, HLCS, HMGB1, HMGCL, HNMT, HNRNPC, HNRNPD, HNRNPH1, HNRNPH2, HNRNPK, HNRNPR, HNRNPU, HOXA1, HPD, HPDL, HPRT1, HRAS, HS2ST1, HSD17B10, HSD17B4, HSPD1, HSPG2, HTRA2, HUWE1, IARS1, IBA57, IDH2, IDS, IDUA, IER3IP1, IFIH1, IFT172, IFT27, IFT74, IGF1, IGF1R, IKBKG, IL1RAPL1, IMPDH2, INPP5E, INPP5K, INTS1, INTS11, INTS13, IQSEC1, IQSEC2, IREB2, IRF2BPL, IRX5, ISCA1, ISCA2, CRPPA, ITFG2, ITPA, ITPR1, ITSN1, IVD, JAM3, JARID2, JMJD1C, KANSL1, KARS1, KAT5, KAT6A, KAT6B, KAT8, KATNB1, KCNA2, KCNA3, KCNB1, KCNC1, KCNC2, KCND2, KCND3, KCNH1, KCNH5, KCNJ10, KCNJ11, KCNJ6, KCNK4, KCNK9, KCNMA1, KCNN2, KCNN3, KCNQ2, KCNQ3, KCNQ5, KCNT1, KCNT2, KCTD3, KCTD7, KDM1A, KDM2A, KDM2B, KDM3B, KDM4B, KDM5A, KDM5B, KDM5C, KDM6A, KDM6B, KATNIP, KIAA0586, BLTP1, KIDINS220, KIF11, KIF14, KIF1A, KIFBP, KIF21B, KIF26A, KIF2A, KIF4A, KIF5A, KIF5C, KIF7, KLF7, KLHL20, KLHL7, KMT2A, KMT2B, KMT2C, KMT2D, KMT2E, KMT5B, KNL1, KPTN, KRAS, L1CAM, L2HGDH, LAMA1, LAMA2, LAMB1, LAMB2, LAMC3, LAMP2, LARGE1, LARP7, LARS1, LARS2, LAS1L, LETM1, LGI3, LHX2, LIAS, LIG4, LINGO4, LINS1, LIPT1, LMBRD1, LMBRD2, LMNB1, LMNB2, LNPK, LONP1, LRP2, LRPPRC, LSS, LTBP1, LYRM7, LZTFL1, LZTR1, MAB21L1, MAB21L2, MACF1, MADD, MAF, MAGEL2, MAN1B1, MAN2B1, MAN2C1, MANBA, MAOA, MAP1B, MAP2K1, MAP2K2, MAP4K4, MAPK1, MAPK8IP3, MAPKAPK5, MAPRE2, MASP1, MAST1, MAST3, MAT1A, MBD5, MBOAT7, MBTPS2, MCCC1, MCM3AP, MCM6, MCOLN1, MCPH1, MDH2, MECP2, MED11, MED12, MED12L, MED13, MED13L, MED17, MED23, MED25, MED27, MEF2C, MEGF8, MEIS2, METTL23, METTL5, MFF, MFSD2A, MFSD8, MGAT2, MICU1, MID1, MIR17HG, MKKS, MKS1, MLC1, MLYCD, MMAA, MMAB, MMACHC, MMADHC, MN1, MOCS1, MOCS2, MOGS, MORC2, MPDU1, MPDZ, MPLKIP, PALS1, MPV17, MRPS22, MRPS34, MSL3, MSMO1, MTFMT, MTHFR, MTHFS, MTO1, MTOR, MTR, MTRR, MTSS2, MMUT, MVK, MYCBP2, MYCN, MYH10, MYO5A, MYT1L, NAA10, NAA15, NAA20, NACC1, NAE1, NAGA, NAGLU, NALCN, NANS, NAPB, NARS1, NBEA, NCAPD2, NCDN, NCKAP1, NDE1, NDP, NDST1, NDUFA1,

NDUFA2, NDUFAF1, NDUFS1, NDUFS4, NDUFS7, NDUFS8, NDUFV1, NEDD4L, NEMF, NEU1, NEUROD2, NEUROG1, NEXMIF, NF1, NFASC, NFIA, NFIB, NFIX, NFU1, NGLY1, NHS, NIPBL, NKAP, NKX2-1, NLGN3, NLGN4X, NONO, NOTCH1, NOVA2, NPC1, NPC2, NPHP1, NPHP3, NR2F1, NR4A2, NRAS, NRCAM, NRROS, NRXN1, NSD1, NSD2, NSDHL, NSRP1, NSUN2, NSUN6, NT5C2, NTNG2, NTRK1, NTRK2, NUBPL, NUDT2, NUP188, NUP214, NUS1, OCLN, OCRL, ODC1, OFD1, OGDH, OGDHL, OGT, OPA3, OPHN1, OSGEP, OTC, OTUD5, OTUD6B, OTX2, OXR1, P4HTM, PABPC1, PACS1, PACS2, PAFAH1B1, PAH, PAK1, PAK3, PAM16, PAN2, PARN, PARP6, PAX5, PAX6, PAX8, PBX1, PC, PCCA, PCCB, PCDH12, PCDH19, PCDHGC4, PCGF2, PCNT, PCYT2, PDE10A, PDE4D, PDE6D, PDGFRB, PDHA1, PDHB, PDHX, PDP1, PDSS1, PDSS2, PDZD8, PEPD, PET100, PEX1, PEX10, PEX11B, PEX12, PEX13, PEX14, PEX16, PEX19, PEX2, PEX26, PEX3, PEX5, PEX6, PEX7, PGAP1, PGAP2, PGAP3, PGK1, PGM2L1, PGM3, PHACTR1, PHF21A, PHF5A, PHF6, PHF8, PHGDH, PHIP, PI4K2A, PI4KA, PIBF1, PIDD1, PIGA, PIGB, PIGC, PIGG, PIGH, PIGK, PIGL, PIGN, PIGO, PIGP, PIGQ, PIGS, PIGT, PIGU, PIGV, PIGW, PIK3C2A, PIK3CA, PIK3R2, PIP5K1C, PISD, PITRM1, PLA2G6, PLAA, PLCB1, PLK1, PLK4, PLP1, PLPBP, PLXNA1, PMM2, PMPCA, PMPCB, PNKP, PNPLA6, PNPT1, POGZ, POLA1, POLG, POLR1A, POLR1C, POLR2A, POLR3A, POLR3B, POLRMT, POMGNT1, POMGNT2, POMK, POMT1, POMT2, PORCN, POU3F2, POU3F3, PPFIBP1, PPIL1, PPM1D, PPP1CB, PPP1R12A, PPP1R21, PPP2CA, PPP2R1A, PPP2R5C, PPP2R5D, PPP3CA, PPT1, PQBP1, PRDM13, PRICKLE2, PRKAR1A, PRKAR1B, PRMT7, PRODH, PRPF8, PRPS1, PRR12, PRUNE1, PSAP, PSMC3, PSMD12, PSPH, PTCD3, PTCH1, PTCHD1, PTDSS1, PTEN, PTF1A, PTPN11, PTPN23, PTPN4, PTRHD1, PTS, PUF60, PUM1, PURA, PUS1, PUS3, PUS7, PYCR1, PYCR2, QARS1, QDPR, QRICH1, RAB11A, RAB11B, RAB18, RAB23, RAB39B, RAB3GAP1, RAB3GAP2, RABGAP1, RAC1, RAC3, RAD21, RAF1, RAI1, RALA, RALGAPA1, RAP1B, RAP1GDS1, RARB, RARS1, RARS2, RBBP8, RBL2, RBM10, RBSN, RELN, RERE, RFT1, RFX3, RFX4, RFX7, RHEB, RHOBTB2, RIT1, RLIM, RMND1, RNASEH2A, RNASEH2B, RNASEH2C, RNASET2, RNF113A, RNF125, RNF13, RNF220, RNU4ATAC, RNU7-1, ROBO1, ROGDI, ROR2, RORA, RPGRIP1L, RPH3A, RPIA, RPL10, RPS6KA3, RRM2B, RSRC1, RTEL1, RTN4IP1, RTTN, SAMD9, SAMHD1, SARS1, SARS2, SART3, SATB1, SATB2, SBF1, SC5D, SCAF4, SCAMP5, SCAPER, SCN1A, SCN1B, SCN2A, SCN3A, SCN8A, SCO2, SDCCAG8, SDHA, SDHAF1, SEMA6B, SEPSECS, SERAC1, SET, SETBP1, SETD1A, SETD1B, SETD2, SETD5, SFXN4, SGPL1, SGSH, SHANK1, SHANK2, SHANK3, SHH, SHMT2, SHOC2, SIAH1, SIK1, SIL1, SIN3A, SIN3B, SIX3, SKI, SLC12A2, SLC12A5, SLC12A6, SLC13A5, SLC16A2, SLC17A5, SLC18A2, SLC19A3, SLC1A1, SLC1A2, SLC1A4, SLC25A1, SLC25A12, SLC25A15, SLC25A22, SLC2A1, SLC30A9, SLC32A1, SLC33A1, SLC35A1, SLC35A2, SLC35C1, SLC38A3, SLC39A14, SLC39A8, SLC46A1, SLC4A10, SLC4A4, SLC5A6, SLC6A1, SLC6A19, SLC6A3, SLC6A8, SLC6A9, SLC9A6, SLF2, SLITRK2, SLX4, SMAD4, SMARCA2, SMARCA4, SMARCA5, SMARCB1, SMARCC2, SMARCD1, SMARCE1, SMC1A, SMC3, SMC5, SMG8, SMG9, SMOC1, SMPD1, SMPD4, SMS, SNAP25, SNAP29, SNAPC4, SNRPB, SNX14, SNX27, SOD1, SON, SOS1, SOS2, SOX10, SOX11, SOX2, SOX4, SOX5, SOX6, SOX9, SPART, AFG2A, AFG2B, SPECC1L, SPEN, SPG11, SPOP, SPR, SPRED1, SPRED2, SPTAN1, SPTBN1, SPTBN2, SPTBN4, SRCAP, SRD5A3, SRRM2, SRSF1, SSR4, ST3GAL3, ST3GAL5, STAG1, STAG2, STAMBP, STIL, STRA6, STRADA, STT3A, STX1A, STX1B, STXBP1, SUCLA2, SUCLG1, SUFU, SUMF1, SUOX, SUPT16H, SURF1, SUZ12, SVBP,

SYN1, SYNCRIP, SYNGAP1, SYNJ1, SYP, SYT1, SZT2, TAF1, TAF2, TAF4, TAF6, TAF8, TANC2, TANGO2, TAOK1, TARS2, TASP1, TAT, TAFAZZIN, TBC1D20, TBC1D23, TBC1D24, TBC1D2B, TBCD, TBCE, TBCK, TBL1XR1, TBR1, TBX1, TCEAL1, TCF20, TCF4, TCF7L2, TCN2, TCTN1, TCTN2, TCTN3, TDP2, TECPR2, TEFM, TELO2, TENM3, TERT, TET3, TFE3, TGIF1, TH, THOC2, THOC6, THRA, THUMPD1, TIAM1, TIMM50, TINF2, TLK2, TMCO1, TMEM106B, TMEM147, TMEM163, TMEM165, TMEM216, TMEM218, TMEM222, TMEM237, TMEM240, RXYLT1, TMEM63B, TMEM63C, TMEM67, TMEM70, TMEM94, TMTC3, TMX2, TNPO2, TNR, TNRC6B, TOE1, TOGARAM1, TP73, TPP1, TPP2, TRA2B, TRAF7, TRAIP, TRAK1, TRAPPC10, TRAPPC11, TRAPPC12, TRAPPC4, TRAPPC6B, TRAPPC9, TREX1, TRIM8, TRIO, TRIP12, TRIT1, TRMT1, TRMT10A, TRNT1, TRPM3, TRRAP, TSC1, TSC2, TSEN15, TSEN2, TSEN54, TSFM, TSHB, TSPOAP1, TTC19, SKIC3, TTC5, TTC8, TTI1, TTI2, TUBA1A, TUBB, TUBB2A, TUBB2B, TUBB3, TUBB4A, TUBG1, TUBGCP2, TUBGCP6, TUSC3, TWIST1, U2AF2, UBA5, UBAP2L, UBE2A, UBE3A, UBE3B, UBE3C, UBE4A, UBR1, UBR7, UBTF, UFC1, UFM1, UFSP2, UGDH, UGP2, UMPS, UNC13A, UNC79, UNC80, UPB1, UPF3B, USP18, USP7, USP9X, VAMP2, VARS1, VARS2, VIPAS39, VLDLR, VPS11, VPS13B, VPS16, VPS33B, VPS41, VPS4A, VPS53, VRK1, WAC, WARS1, WARS2, WASF1, WASHC4, WBP4, WDFY3, WDPCP, WDR11, WDR26, WDR37, WDR4, WDR45, WDR45B, WDR5, WDR62, WDR73, WDR81, WIPI2, WNK3, WNT1, WNT5A, WWOX, XPA, XRCC4, XYLT1, YARS1, YIF1B, YWHAE, YWHAG, YY1, ZBTB11, ZBTB18, ZBTB20, ZBTB24, ZBTB7A, ZC4H2, ZDHHC9, ZEB2, ZFHX4, ZFYVE26, ZIC1, ZIC2, ZMIZ1, ZMYM2, ZMYM3, ZMYND11, ZMYND8, ZNF142, ZNF148, ZNF292, ZNF335, ZNF462, ZNF526, ZNF699, ZNF711, ZNHIT3, ZSWIM6, DMPK

Panel of 1035 genes associated with neuromuscular disorder
AARS1, ABAT, ABCA1, ABCB7, ABCD1, ACADM, ACADVL, ACO2, ACOX1, ACP5, ACTA1, ACTB, ACTN2, ADAR, ADCY5, ADCY6, AFG3L2, AGL, AGRN, ALDH3A2, ALDH5A1, ALDOA, ALS2, SETX, AMPD2, ANG, RIPK4, TRPA1, ANXA11, AP1S2, AP4B1, AP4E1, AP4M1, AP4S1, APP, AR, ARG1, ARL6IP1, ARSA, ASAH1, ASPA, ZFHX3, ATCAY, ATM, ATP1A1, ATP1A2, ATP1A3, ATP2A1, ATP5MC3, ATP6V0A1, ATP6AP1, ATP7A, ATP7B, KIF1A, AUH, B3GAT3, B4GALT1, B4GALT7, BAG3, BBS1, BCKDHB, BCS1L, BIN1, DST, TWNK, DAGLA, GIPC1, SAMD9L, ERLIN2, CA8, CACNA1A, CACNA1E, CACNA1G, CACNA1S, CACNA2D2, CACNB4, CAD, CAMK4, CAPN1, CAPN3, CASK, CAV3, MICU1, CCNF, CD59, CENPF, CFL2, VPS13A, CHAT, CHKB, CHRNA1, CHRNB1, CHRND, CHRNE, CHRNG, LYST, CHST3, CLCN1, CLCN2, TPP1, CLN3, CLN5, CLN6, CLPP, SBF2, VPS13B, COL12A1, COL13A1, COL4A1, COL4A2, COL6A1, COL6A2, COL6A3, COLQ, COX10, COX15, COX6A1, COX6A2, CP, CPOX, CPT2, CRLF1, MED27, CRYAB, CSF1R, CSTB, CTBP1, CTDP1, CTSF, OFD1, CYP27A1, CYP7B1, DAB1, DAG1, DARS1, DBH, DCTN1, DDB2, DDC, DHX9, DES, DGUOK, DHCR24, DHCR7, DHFR, DKC1, DLAT, DLD, DMD, DMPK, DYNC1H1, DNM2, DNMT1, DOCK3, DPAGT1, DPM1, DPM2, DPM3, DPYD, DRP2, ATN1, DYSF, TOR1A, EBP, ECEL1, TYMP, ECHS1, EGR2, EIF2B1, EIF2B2, EIF2B3, EIF2B4, EIF2B5, EMD, ENO3, ENTPD1, EPM2A, EPRS1, ERBB3, ERBB4, ERCC4, ERCC5, ERCC6, ERCC8, ETFA, ETFB, ETFDH, EVC, EXT1, EXT2, FAH, FARSA, FBLN5, FBN2, FKTN, FGF14, FGFR2, FGFR3, FHL1, FLNA, FLNB, FLNC, FMR1, FOLR1, FOXG1, FXN, FTL, FUCA1, FUS, FUT8, FXR1, ISG15, SLC37A4, GAA, GALC, B4GALNT1, GALNT2, GALNT3, GAN, GARS1, GBA1, GBE1, GBF1, GCDH, GCH1,

BLOC1S1, GFAP, GFPT1, GGPS1, GJA1, GJB1, GLA, GLB1, GLE1, GLI3, GLRA1, GLRB, GLS, GM2A, GNAL, GNAO1, GNB1, GOSR2, GPAA1, ADGRG1, GRID2, GRIN1, GRM1, GRN, GSN, GTPBP2, GYG1, GYS1, HADHA, HADHB, HARS1, HCFC1, HTT, HEXA, HEXB, HIBCH, HINT1, HK1, HMBS, HMGCL, HNRNPA1, HNRNPA2B1, HNRNPDL, HNRNPH1, HPCA, HPRT1, HSD17B4, DNAJB2, HSPB1, HSPD1, HSPG2, IGHMBP2, ELP1, IMPDH2, IRF6, ITGA7, STT3A, ITM2B, ITPR1, ITPR3, IVD, JAG1, JAG2, KCNA1, KCNA2, KCNC3, KCND3, KCNJ10, KCNJ2, KCNMA1, KCNN2, KCNQ2, KIF1C, KIF5A, KPNA3, L1CAM, LAMA1, LAMA2, LAMB1, LAMP2, LARGE1, LDHA, COG1, LIG3, LMNA, LMNB1, LMOD3, LMX1B, LRP4, SMAD3, SMAD4, MAG, MAGEL2, MAN1B1, MAOA, MAPK8IP3, MAPT, MARS1, MATR3, CHST6, MCM3AP, MEF2C, MGAT2, MINPP1, ATXN3, MKS1, MME, MPDU1, MPI, MPV17, MPZ, MRE11, SEPTIN9, MT-ATP6, MT-ATP8, MTHFR, MTM1, MTMR2, MT-ND1, MT-ND6, MTTP, MT-RNR1, MT-TL1, MUSK, MMUT, MVK, MYBPC1, MYH2, MYH3, MYH7, MYH8, MYL1, MYL2, MYO9A, MYOD1, NAGA, NDRG1, NDUFA1, NDUFA10, NDUFA2, NDUFS1, NDUFS2, NDUFS4, NDUFS7, NDUFS8, NDUFV1, NEB, NEFH, NEFL, NEK1, NGF, NOTCH3, NPC1, NPHP1, NPHP3, CNTNAP1, NT5C2, NTRK1, OCLN, OCRL, OPA1, OPA3, OPHN1, SIGMAR1, CLDN11, PABPN1, PAFAH1B1, PAH, PRKN, PAX6, PAX7, PCCA, PCCB, CHMP1A, PCYT2, AIFM1, PDE10A, PDE2A, PDGFB, PDGFRB, PDHA1, PDK3, PDYN, PEX1, PEX10, PEX11B, PEX12, PEX13, PEX14, PEX16, PEX3, PEX6, PEX7, PFKM, PFN1, PGAM2, PGK1, PGM1, PGM3, PHKA1, PHYH, PIGA, PIGB, PIGL, PIGN, PI4KA, PLA2G6, PLEC, PLOD1, PLOD2, PLP1, PMM2, PMP2, PMP22, PMPCB, EXOSC9, PHOX2B, PNKD, PNKP, POLG, POLG2, POMT1, POR, POU4F1, CTSA, PDP1, PPOX, PPP2R2B, PPP2R5D, PRDX3, PRG4, PRKAG2, PRKCG, EIF2AK2, PRKRA, DNAJC3, PRNP, PRPS1, HTRA1, PSAP, PSEN1, PSEN2, PTEN, HACD1, PTPN11, CAVIN1, PTS, PEX19, PEX2, PEX5, ALDH18A1, PYGM, QDPR, RAB11B, RAB7A, RAPSN, RARS1, RELN, RFC1, RORA, RPIA, RPS6KA3, RTN2, RYR1, RYR3, SACS, SARS1, SBF1, ATXN1, ATXN10, ATXN2, ATXN7, ATXN8OS, SCN10A, SCN11A, SCN1A, SCN4A, SCN8A, SCN9A, SCO1, SCO2, SCP2, AIMP1, SDHA, SDHB, SEC23B, SGCA, SGCB, SGCD, SGCE, SGCG, ST3GAL5, SKI, SLC12A6, SLC16A2, SLC17A5, SLC18A2, SLC18A3, SLC1A3, SLC1A4, SLC20A2, SLC22A5, SLC25A1, SLC25A12, SLC25A15, SLC25A4, SLC2A1, SLC35A1, SLC35A2, SLC5A6, SLC6A3, SLC6A8, SLC6A9, SLC9A1, SLC9A6, KDM5C, SMN1, SNAP25, SNCA, SOD1, SORD, SOX10, SPG11, ATL1, SPAST, SPG7, SPR, SPTAN1, SPTBN2, SPTLC1, SPTLC2, SQSTM1, SSR4, STIM1, STUB1, SUCLA2, SUCLG1, SUOX, SURF1, SYNGAP1, SYNJ1, SYT2, TAF1, BRF1, TARDBP, TBK1, TBP, TCAP, TERT, TFG, TGFB2, TGFB3, TGFBR1, TGFBR2, TH, TIA1, TIMM8A, TINF2, NKX2-1, TK2, TNNC2, TNNI2, TNNT1, TNNT3, MED12, TPM2, TPM3, TREX1, TRIP4, TSFM, CEP41, MYOT, TTN, TTPA, TTR, TUFM, TYROBP, UBAP1, UBA1, UBQLN2, UBTF, UCHL1, USP18, VAMP1, VAMP2, VAPB, VCP, VLDLR, VPS33B, VPS41, VRK1, WARS1, WARS2, WFS1, WWOX, XK, XPA, XPC, XPR1, XRCC1, YARS1, YY1, ZMPSTE24, CNBP, ADAMTS10, LPIN1, MCOLN1, ROBO3, VPS35, VPS4A, RXYLT1, ATP8A2, FBXO7, FBXL4, MACF1, AAAS, DEGS1, GFM1, PRX, ADGRG6, FOXP2, ABHD16A, PRDM12, PRDM13, ANO3, SLC5A7, CCDC78, JPH3, CIC, IRF2BPL, BCAS3, HTRA2, SCYL1, SLC25A19, ELOVL4, ELOVL1, TMEM237, NPC2, WNK1, PINK1, VPS11, VPS16, COG5, DNAJB6, SPTBN4, PIGS, PIGT, PUM1, SNX14, DNAJC6, TSEN34, XYLT1, XYLT2, JAM3, CHCHD10, KLHL7, ALG9, B4GAT1, LDB3, LRPPRC, BSCL2, KMT2B, RBCK1, ABHD12, PANK2, NDUFAF5, NOP56, SAMHD1, TOE1, GDAP1, TRIM2, APTX, SELENON,

MPLKIP, ACER3, FITM2, SLC52A3, DNAJC5, TGM6, SLC19A3, PNPLA6, NFU1, ADAMTS15, PARK7, TRIM32, RAB39B, BCAP31, EDEM3, COQ8A, LITAF, FIG4, MFN2, SPEG, KLHL41, ATG7, ERLIN1, CLP1, EXOSC8, NIPA1, MLC1, SYNE1, LARS2, TNPO3, OPTN, CHSY1, BICD2, AGTPBP1, RRM2B, TAF8, TPK1, GJC2, KAT6B, NGLY1, PITRM1, TREM2, TDP2, MAP3K20, FARSB, NUP188, PGAP2, EXOSC3, REEP2, B3GALT6, FKRP, GPT2, TRPV4, TMEM199, MYO18B, FKBP10, ALG1, ASXL1, FICD, NAXE, ATP6V0A2, SPART, RNASEH2A, CPT1C, NEK9, LRRK2, COG4, COG6, COG7, COG8, FKBP14, PMPCA, CRB2, ASCC3, LGI4, SLC44A1, CAMTA1, NDUFAF1, IFIH1, GBA2, NALCN, EBF3, FGD4, POMGNT1, TTBK2, SCAF4, NKX6-2, ALG12, MECR, DDHD1, POMT2, EVC2, ISCA2, SCARF2, MYORG, KIAA0586, GEMIN5, ADSS1, FLVCR2, GLRX5, POLR1C, SLC35C1, B3GLCT, NUBPL, VIPAS39, SPG21, SUMF1, COA8, L2HGDH, CYP2U1, UFM1, DHDDS, DPYSL5, TXNDC15, ARMC9, GNB4, ZFYVE26, TUBA1A, TUBB3, TUBB4A, SLC35D1, THAP1, SLC39A14, SLC39A8, AARS2, HACE1, SERAC1, FARS2, FA2H, CILK1, ADPRS, PDHX, RARS2, INPP5E, ACAD9, AHI1, NHLRC1, CHCHD2, RNASET2, RNF216, ANTXR2, VMA21, FAM20C, AP5Z1, TMEM106B, GMPPA, GMPPB, PEX26, ALG3, SLC29A3, ALG6, ALG2, ALG8, PNPT1, PIGW, PIGO, UBA5, MYPN, ETHE1, ACBD5, PIBF1, DOLK, MORC2, VPS13D, GNE, PGAP3, PTF1A, TMEM63C, INF2, PACS2, NDUFA12, RNASEH2C, BEAN1, PTRH2, ASCC1, CSGALNACT1, TACO1, BOLA3, CHST14, TCTN3, MMACHC, ATL3, CHMP2B, C2CD3, HYCC1, NRROS, SIL1, FLVCR1, G6PC3, MOGS, CEP104, ZC4H2, TMEM216, WDR45B, MARS2, LRSAM1, TMEM240, SLC25A46, COQ2, NSRP1, SLC30A10, RNF170, DDX59, ARL13B, TANGO2, C19orf12, VAC14, ANO10, DARS2, RNF220, ATAD3A, NAXD, OGDHL, VPS53, CWF19L1, TBC1D23, RNASEH2B, GORAB, KCTD17, COA7, TRAPPC11, TCTN2, DCAF17, REEP1, CPLANE1, SRD5A3, ORAI1, POMGNT2, WDR73, RETREG1, TTC19, PIGV, TCTN1, PYROXD1, CTC1, COLGALT1, CSPP1, STN1, FAR1, POMK, PIEZO2, HEPACAM, SCLT1, HYLS1, DOK7, WDR81, MTRFR, FOXRED1, HIKESHI, TMEM138, BLTP1, COX20, IBA57, ANO5, TMEM218, TSEN54, LYRM7, NDUFAF2, TMEM107, CCDC115, HPDL, ALG14, C9orf72, D2HGDH, TMEM67, TSEN2, STAC3, EOGT, B3GALNT2, NDUFAF6, B9D2, ISCA1, AFG2B, MAGT1, DNAJC12, WDR45, WASHC5, CEP290, SMCHD1, PLEKHG5, DDHD2, KIAA0753, TMEM63A, MTCL1, RPGRIP1L, ERGIC1, CC2D2A, ZSWIM6, EPG5, EARS2, SH3TC2, TOR1AIP1, KIDINS220, GLDN, MEGF10, MTFMT, MSTO1, IARS2, HECW2, NFASC, ISCU, NDUFAF3, COASY, POLR3A, HSPB8, ATP13A2, RFT1, SLC52A2, TUSC3, PYCR2, POLR3B, KLHL40, KIF7, PRRT2, YIF1B, DNAJC19, FDX2, SEPSECS, CLPB, TARS2, TMEM165, TUBB2B, VWA1, LRP12, CFAP276, ALG11, SMPD4, SNORD118, MYMK, PTRHD1, SDHAF1, INPP5K, RNU7-1, KBTBD13, TMEM231, CRPPA, PET100

GRCh37 STR panel for NDD
AFF2, CNBP, DMPK, EIF4A3, FMR1, XYLT1, ZIC2

GRCh37 STR panel for NMD
AR, ATNI, ATXNI, ATXNIO, ATXN2, ATXN3, ATXN7, ATXN8OS, BEAN1, C9orf72, CACNA1A, CNBP, CSTB, DABI, DMPK, FGF14, FMR1, FXN, GIPC1, GLS, HTT, JPH3, LRP12, NOP56, PABPN1, PHOX2B, PPP2R2B, RFC1, TBP, THAP11, XYLT1, ZFHX3

GRCh38 STR panel

ABCD3, AFF2, AR, ARX_EIEE, ATN1, ATXN10, ATXN1, ATXN2, ATXN3, ATXN7, ATXN8OS, C9ORF72, CACNA1A, CBL, BEAN1, CNBP, CSTB, DAB1, DIP2B, DMPK, EIF4A3, FMR1, FGF14, FOXL2, FXN, HOXD13, HTT, JPH3, LRP12, GIPC1, NOP56, NOTCH2NLC, PABPN1, PHOX2B, PPP2R2B, RAPGEF2, RFC1, RILPL1, RUNX2, SAMD12, SOX3, TBP, TBX1, TCF4, THAP11, TNRC6A, XYLT1, ZIC2, ZIC3, GLS, ZFHX3

Document S2: Differential methylation calling of Oxford Nanopore Technologies data

Differential methylation was called using ESS (https://github.com/J35P312/ESS). The script takes methylartist phased DSS files as input and converts these files into a bed file for each individual. The script is run for cases and controls.

Bash convert_bed.sh {prefix}

Where prefix is the prefix used while running methylartist. Next a textfile is made, describing the absolute file path to each of the control samples. Lastly, ESS is run using the following command:

Python ess.py {case}.bed.gz controls.txt praderwilli.txt

Where case is the prefix of the case being run, controls.txt is the text file listing the path to each control sample, and praderwilli.txt is a bed file listing the regions of interest.
Briefly, the script computes the median faction of methylated bases in haplotype 1 and haplotype 2 for the case, a skewness metric is obtained by computing the absolute difference between these two medians.
Next the same metric is obtained for each control sample.
Lastly, the case is compared to the controls through a variety of statistics, including a 95% confidence interval according to a t-distribution, as well as a Z score. These statistics are then printed to stdout in as a comma separated table.
