# Supplementary material for: Long-read genome sequencing enhances diagnostics of pediatric neurological disorders
Source: Genome Med. 2026 Jan 9;18:12. doi: 10.1186/s13073-025-01596-5 (PMC12838436; doi:10.1186/s13073-025-01596-5)
Supplement: Supplementary file 3 — Supplementary Material 3. [file 13073_2025_1596_MOESM3_ESM.pdf]

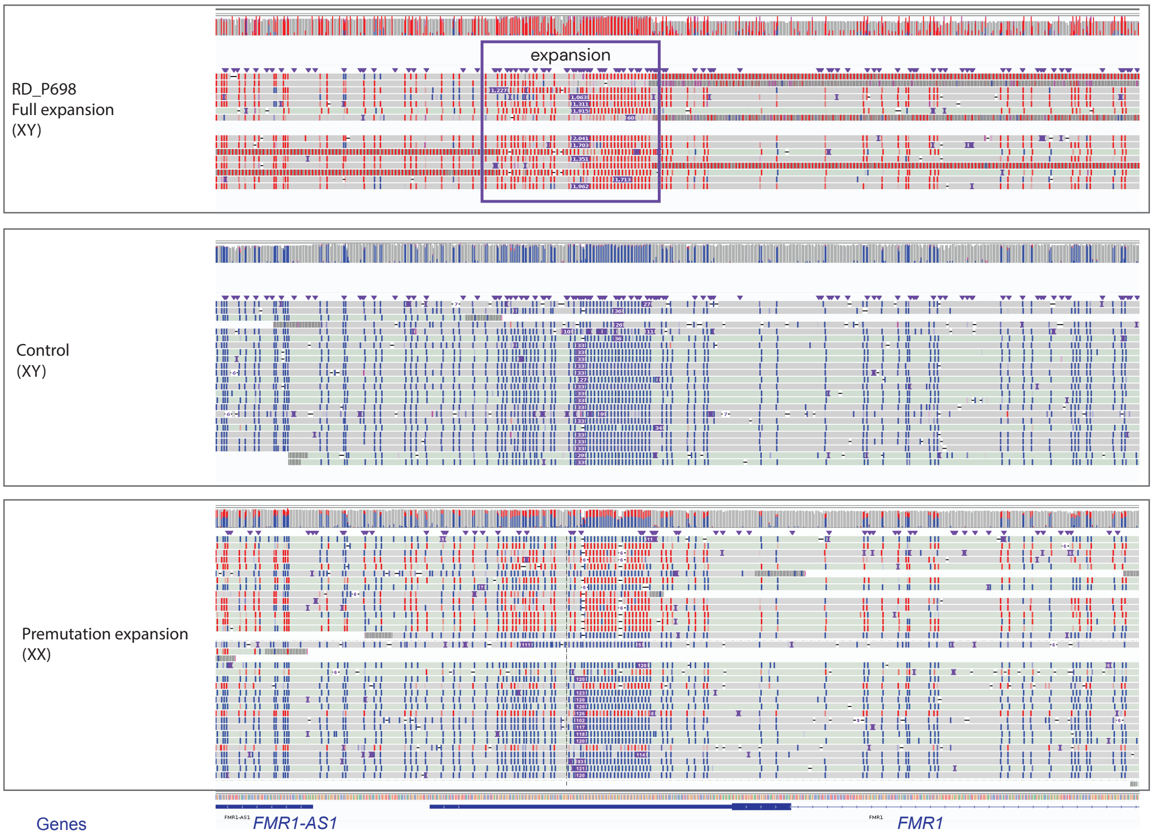

Fig. S1. Methylation pattern across *FMR1*. The integrative genomics viewer (IGV) screenshots showing the beginning and upstream region of *FMR1* with methylated CpGs (indicating inactive promotor) in red and unmethylated CpGs (indicating active promotor) in blue. Top: A male with a full expansion, visualized as purple boxes marking the insertion or as long repetitive reads extending beyond the view (likely because they do not span the entire expansion). The CpGs shows hypermethylation across the region. Middle: A male control displays hypomethylation. Bottom: A female carrier of a premutation-range expansion, one allele is expanded (purple box) and the other is normal. The expanded allele is predominantly hypomethylated, while the normal allele is predominantly hypermethylated.

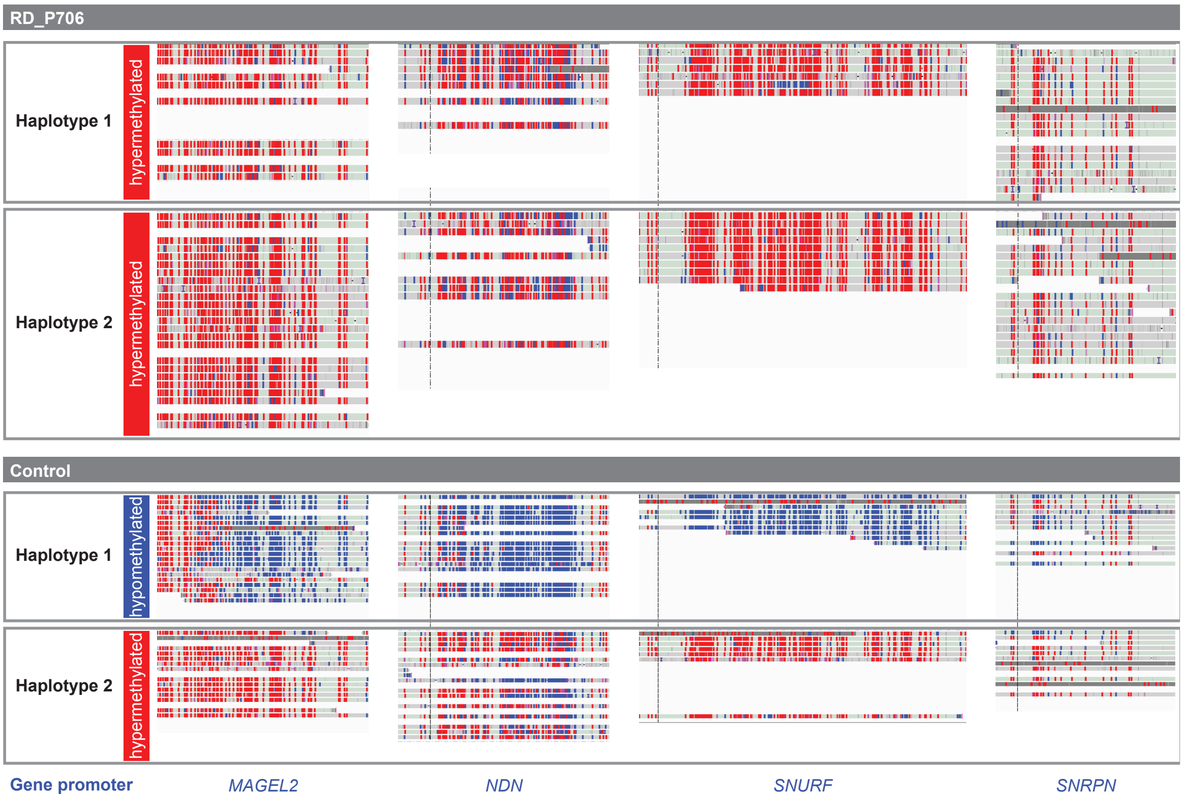

Fig. S2. Methylation pattern across promoter regions of genes in the Prader-Willi region. Integrative genomics viewer (IGV) screenshots with phased reads spanning the promoter regions of *MAGEL2*, *NDN*, *SNURF* and *SNRPN*, showing methylated CpGs in red and unmethylated CpGs in blue. The individual with maternal uniparental disomy of chromosome 15 (top) shows hypermethylation across all four gene promoter regions on both haplotypes. In a control individual (bottom), the methylation pattern shows haplotype 1 as hypomethylated (paternal) and haplotype 2 as hypermethylated (maternal).

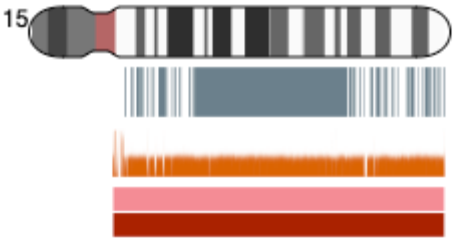

Fig. S3. Maternal uniparental disomy. Trio-based short-read genome sequencing showing maternal uniparental isodisomy across a segment of chromosome 15. The chromosome 15 ideogram is shown at the top. Homozygous single-nucleotide variants (SNVs) in the proband are indicated in gray, forming a contiguous block if consistent with loss of heterozygosity. Read-depth across the chromosome is shown in orange, demonstrating no major copy-number change in the affected region. Haplotype assignment based on parental genotypes is shown below, demonstrating that SNVs within the affected region correspond to the maternal haplotype shown in pink and red. SNVs of paternal origin would be indicated in yellow or blue.

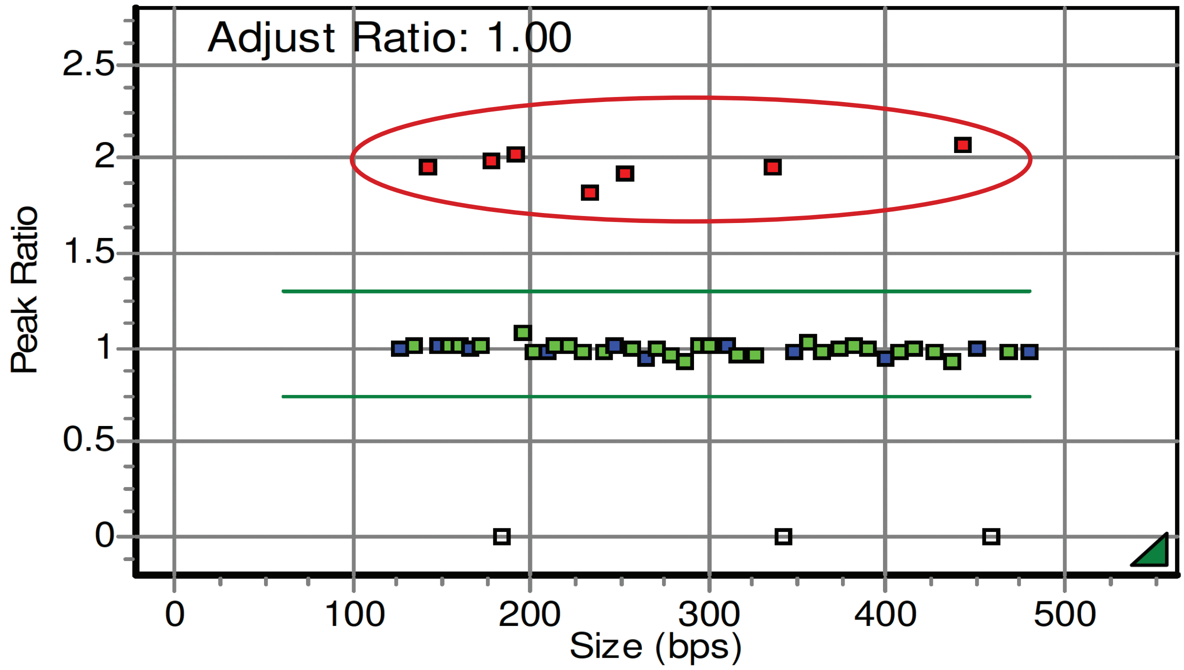

Fig. S4. MLPA results of the SMN region in RD_P623. The red probes (red circle) correspond to methylation across *SNRPN*.

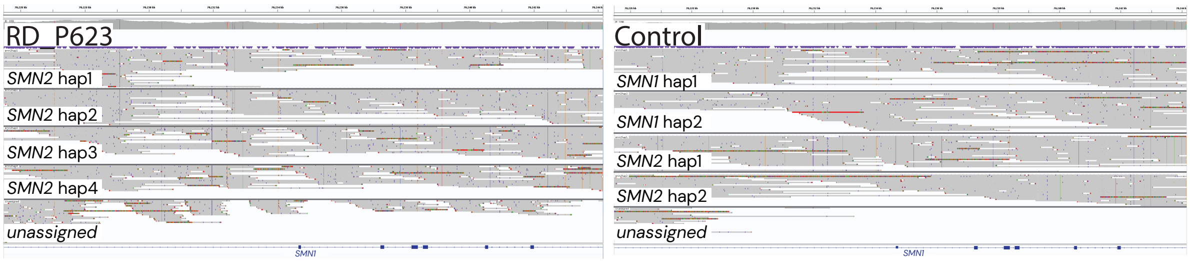

Fig. S5. Phasing of *SMN1* and *SMN2*. Integrative genomics viewer (IGV) screenshot across the *SMN1* region. In individual with homozygous deletion of *SMN1* (RD_P623), no haplotypes corresponding to *SMN1* are observed, while four haplotypes correspond to *SMN2*. In the control individual, there are two haplotypes identified as *SMN1* and two as *SMN2*.

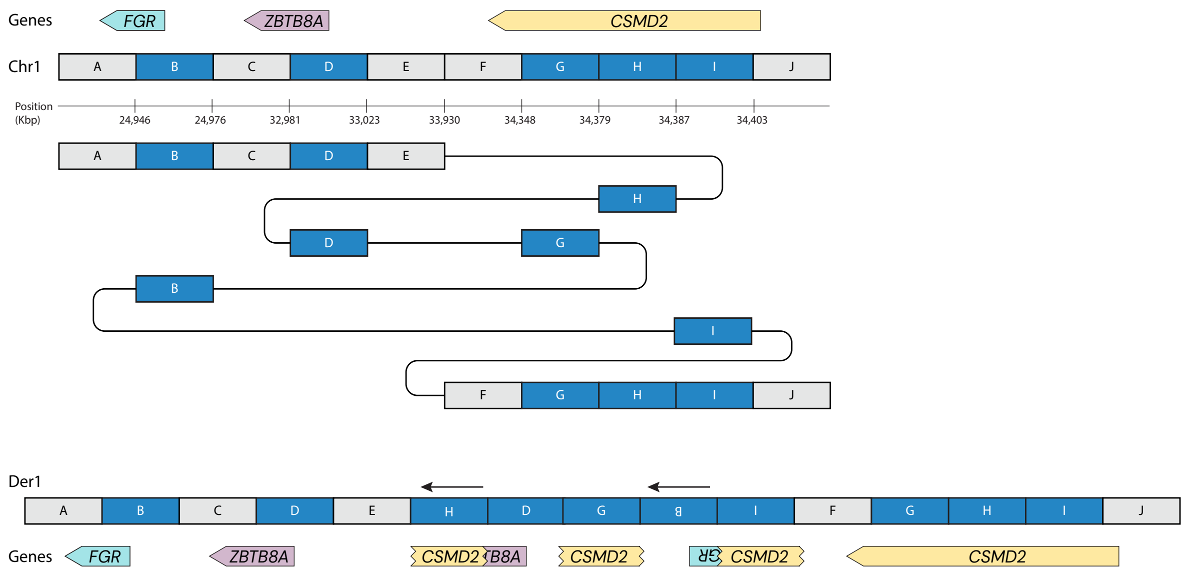

Fig. S6. Chromoanasynthesis event of chromosome 1p. Schematic of the reference chromosome 1 across the affected region, with duplicated segments shown in blue, and genes at breakpoints displayed above. Middle: Subway plot of the rearrangement, illustrating duplicated segments inserted between segments E and F in a disorganized manner. Bottom: Resolved derivative structure, with inverted segments indicated by arrows. Below, a depiction of the genes involved in the rearrangement.

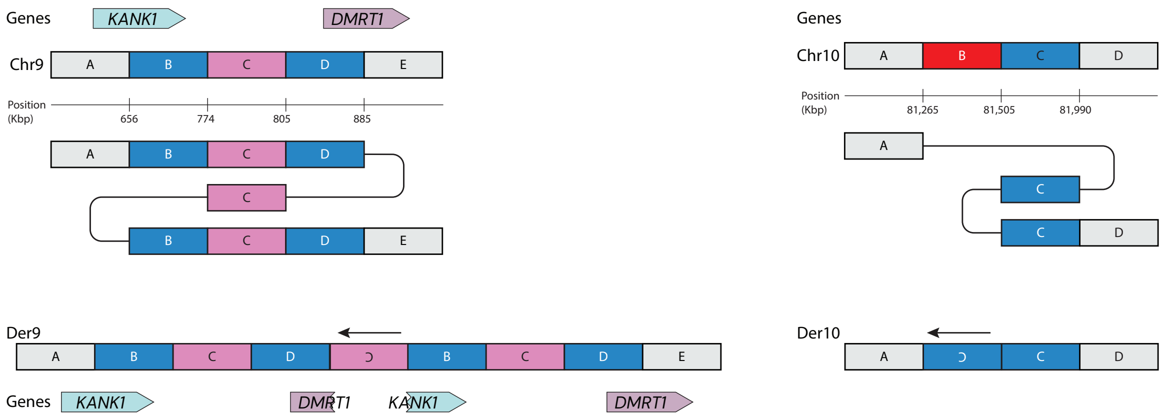

Fig. S7. Complex SVs on chromosomes 9p and 10p. Top: Schematic of the reference chromosome 9 and 10 across the affected region, with deleted segments (red), duplicated segments (blue) and triplicated segments (pink). Genes at breakpoints displayed above. Middle: Subway plot of the rearrangements, showing a DUP-INV/TRIP-DUP and DEL-INV-DUP structure, respectively. Bottom: Resolved derivative structures with inverted segments indicated by arrows. Below, a depiction of the genes involved in the rearrangement.
